# Ultrasound-Enhanced Gelation of Stimuli-Responsive and Biocompatible Phenylalanine-Derived Hydrogels

**DOI:** 10.3390/gels11030160

**Published:** 2025-02-23

**Authors:** Eduardo Buxaderas, Yanina Moglie, Aarón Baz Figueroa, Juan V. Alegre-Requena, Santiago Grijalvo, César Saldías, Raquel P. Herrera, Eugenia Marqués-López, David Díaz Díaz

**Affiliations:** 1 Instituto de Química del Sur (CONICET-UNS), Departamento de Química, Universidad Nacional del Sur, Av. Alem 1253, Bahía Blanca 8000, Argentina; ebuxaderas@gmail.com (E.B.); yamoglie@ull.edu.es (Y.M.); 2 Instituto Universitario de Bio-Orgánica Antonio González, Universidad de La Laguna, Avda. Astrofísico Francisco Sánchez 2, 38206 La Laguna, Spain; alu0100897234@ull.edu.es; 3 Departamento de Química Orgánica, Universidad de La Laguna, Avda. Astrofísico Francisco Sánchez 3, 38206 La Laguna, Spain; 4 Departamento de Química Inorgánica, Instituto de Síntesis Química y Catálisis Homogénea (CSIC-University of Zaragoza), C/Pedro Cerbuna 12, 50009 Zaragoza, Spain; jv.alegre@csic.es; 5 CIBER-BBN, ISCIII, Jordi Girona 18-26, 08034 Barcelona, Spain; sgrgma@cid.csic.es; 6 Departamento de Química Física, Facultad de Química y de Farmacia, Pontifica Universidad Católica de Chile, Santiago 7820436, Chile; casaldia@uc.cl; 7 Departamento de Química Orgánica, Laboratorio de Organocatálisis Asimétrica, Instituto de Síntesis Química y Catálisis Homogénea (CSIC-University of Zaragoza), C/Pedro Cerbuna 12, 50009 Zaragoza, Spain; raquelph@unizar.es; 8 Institute of Organic Chemistry, University of Regensburg, Universitätsstr. 31, 93040 Regensburg, Germany

**Keywords:** supramolecular hydrogel, amino acid, phenylalanine, ultrasound, sonogelation, drug release

## Abstract

Stimuli-responsive materials, particularly supramolecular hydrogels, exhibit a dynamic adaptability to external factors such as pH and ultrasound. Among these, phenylalanine (Phe)-derived hydrogels are promising due to their biocompatibility, biodegradability, and tunable properties, making them ideal for biomedical applications. This study explores the effects of pH and ultrasound on the gelation properties of *N*-substituted Phe derivatives, with a primary focus on the role of ultrasound in optimizing the gelation process. A series of *N*-substituted Phe derivatives were synthesized via reductive amination and hydrolysis. Hydrogel formation was possible with two of these compounds, namely **G1** and **G2**, using the following two methods: heating–cooling (H–C) and heating–ultrasound–cooling (H–US–C). The critical gelation concentration (CGC), gelation kinetics, thermal stability (*T*_gel_), and viscoelastic properties were assessed. Morphological and cytotoxicity analyses were performed to confirm the suitability of these gels for biomedical applications. Both **G1** and **G2** derivatives demonstrated enhanced gelation under the H–US–C protocol compared to H–C, with notable reductions in CGC (up to 47%) and gelation time (by over 90%). Ultrasound-induced gels led to an improved network density and stability, while maintaining thermal reversibility and mechanical properties comparable to those of hydrogels formed without ultrasound. Cytotoxicity studies confirmed a high biocompatibility, with cell viability rates above 95% across the tested concentrations. Given the similar rheological and morphological properties of the hydrogels regardless of the preparation method, drug release experiments were performed with representative gel samples and demonstrated the efficient encapsulation and controlled release of 5-fluorouracil and methotrexate from the hydrogels, supporting their potential as pH-responsive drug delivery platforms. This study highlights the role of ultrasound as a powerful tool for accelerating and optimizing the gelation process of supramolecular hydrogels, which is particularly relevant for applications requiring rapid gel formation. The developed Phe-based hydrogels also demonstrate promising characteristics as drug delivery systems.

## 1. Introduction

Stimuli-responsive materials, often referred to as ‘smart’ or ‘intelligent’ materials, exhibit dynamic changes in their chemical or physical properties when exposed to external stimuli such as pH, temperature, light, electromagnetic fields, or mechanical stress [1,2]. Such a response can be reversible or irreversible, altering important properties (e.g., shape, solubility, and color). The capacity of these systems to dynamically adapt to changing conditions has increased their industrial applications across high-tech fields such as biomedical engineering, environmental remediation, soft robotics, and sensors [3,4].

Among these materials, gels represent a unique class [5,6] due to their semi-solid nature and remarkable ability to hold substantial liquid volumes within the interstitial spaces of a solid network (minor component), enabled by capillary forces or surface tension. From a rheological point of view, gels display a higher storage modulus (*G*′) than loss modulus (*G*′′). A distinction is frequently made between chemical (or polymer) [7] and physical (or supramolecular) gels [8]. The former are formed through covalent bonds between polymer chains, affording permanent gel networks that are typically irreversible (i.e., they do not revert to a liquid phase). In contrast, physical gels are formed by the self-assembly of low-molecular-weight (LMW) compounds (called gelators), which are typically reversible under specific conditions because the network is formed mainly by noncovalent interactions such as hydrogen bonds, electrostatic interactions, van der Waals forces, and coordination or hydrophobic interactions [9]. This reversibility is usually accompanied by a weaker mechanical stability than that of chemical gels. Thus, the nature of the interactions governing the gelation process affects the responsiveness, stability, and application suitability of gel materials. The unique features of gels, such as their high liquid content and viscoelastic properties, have made these materials very attractive for a number of applications across important fields, such as biomedicine, cosmetics, agriculture, environmental remediation, the food industry, confined catalysis, electronics, and soft robotics [10,11,12,13,14].

Within the context of supramolecular gels, those derived from amino acids have gained significant interest due to their inherent biocompatibility, biodegradability, and structural versatility [15]. Among the available libraries of essential amino acids, phenylalanine (Phe) has attracted major attention as a building block owing to its unique structural properties and ability to self-assemble in organic and aqueous media, affording gels that have been used for drug delivery, as an extracellular matrix for tissue engineering, oil spill recovery, the removal of dyes, and the extraction of heavy metals or pollutants, and for the detection of explosives [16].

Conventionally, physical gels are prepared via heating–cooling (H–C) protocols [17], but alternative techniques such as ultrasound-induced gelation (sonogelation) [18,19,20] have emerged, producing materials with enhanced properties through mechanisms involving localized heating, cavitation, high-pressure zones, and shear forces that promote molecular interactions [18,21,22]. As a matter of fact, ultrasound-induced gelation has recently been recognized as a paradigm shift [17].

Herein, we describe the synthesis of a series of *N*-substituted Phe derivatives and their ability to form supramolecular hydrogels in response to external stimuli. Both the effects of pH and preparation method (i.e., H–C vs. heating–ultrasound–cooling (H–US–C)) were studied and compared in terms of gelation kinetics and gel properties.

## 2. Results and Discussion

### 2.1. Synthesis of N-Substituted Phe Derivatives

**Figure 1:**
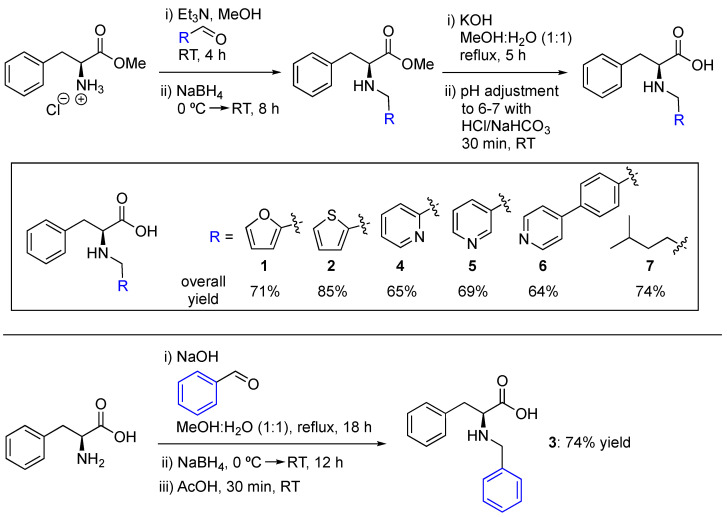
Synthetic routes to Phe derivatives **1**–**7**.

*N*-Substituted Phe derivatives were rapidly synthesized starting from the hydrochloride salt of the corresponding methyl ester by reductive amination and subsequent hydrolysis (Figure 1). The first step was performed at room temperature (RT) in methanol in the presence of triethylamine (TEA) and the desired aldehyde to form a corresponding oily imine intermediate. Then, NaBH_4_ was used as reducing agent to afford the expected amine intermediate within 8 h. After this period, solvent removal and standard extraction were carried out, and the obtained crude was submitted to basic hydrolysis. The desired product was precipitated after 5 h by adjusting the pH to ca. 6–7. Finally, the application of standard solvent removal, filtration, washing, and drying protocols enabled the isolation of the most desired Phe derivatives (compounds **1**, **2**, and **4**–**7**) as white solids in a ca. 64–85% overall yield (Figure 1, *top*). The Phe derivative formed from benzaldehyde (compound **3**) was synthesized by a similar procedure but starting from the corresponding unprotected amino acid, and required the use of NaOH as base and a longer reaction time (Figure 1, *bottom*) (see Experimental Section for detailed procedures). Nuclear magnetic resonance (NMR) spectra, as well as other characterization data of the compounds, are provided in the ESI.

### 2.2. Solubility and Gelation Properties

The gelation ability of the synthesized compounds was investigated via a standard H–C protocol. Materials that did not exhibit gravitational flow upon the inversion of the vial upside-down were classified as gels. Stable hydrogels were obtained from LMW compounds **1** and **2** at pH values between 4.8 and 10.9 (see ESI, Table S2, solutions S3–S6) via the use of universal Britton–Robinson buffer and phosphate-buffered saline (PBS). In contrast, the dissolution of compound **4** at an acidic pH resulted in the formation of well-defined crystals. In contrast, compounds **3**, **5**, **6**, and **7** were partially insoluble over the entire pH range. Therefore, we focused on the study of the hydrogels from compounds **1** and **2** (hereafter referred to as gelator **G1** and gelator **G2**, respectively) and studied the gel formation via the following two different protocols: the standard H–C protocol and H–US–C protocol. While the H–C method consisted of the spontaneous cooling of the isotropic solution to RT, the H–US–C protocol involved a cooling step under the application of ultrasound at RT directly after heating until gel formation. The hydrogels obtained from both protocols were compared in terms of their critical gelation concentration (CGC), gelation kinetics, and thermal stability (*T*_gel_). Notably, all the formed hydrogels were white and opaque in appearance (see ESI, Figure S2), indicating the formation of aggregate structures larger than the wavelength of visible light, independent of the protocol used to induce the gelation. However, the effects of ultrasound on gelation properties are discussed next.

### 2.3. Effect of Ultrasound on Gelation Ability and Critical Gelation Concentration

**Figure 2:**
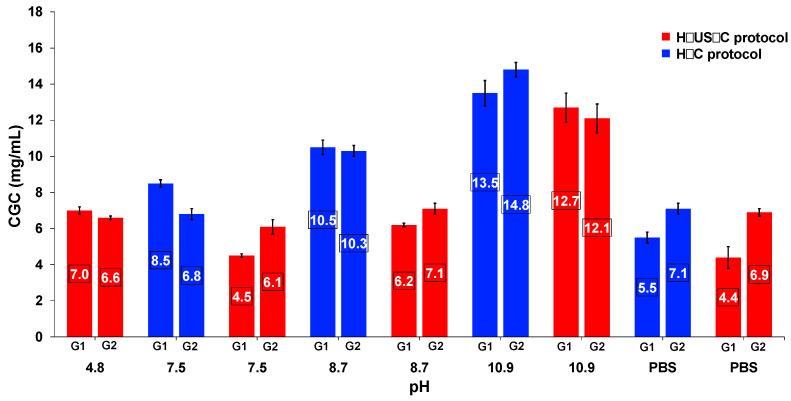
Comparison of the critical gelation concentrations of **G1** and **G2** at different pH values using the H–US–C protocol and H–C protocol.

The use of the H–US–C protocol for hydrogel formation using **G1** and **G2** allowed gelation at pH 4.8 (S3), whereas, when the H–C protocol was used, no gelation was observed. In the pH range between 7.5 (S4) and 10.9 (S6) with PBS (pH = 7.4) as the solvent, the H–US–C protocol caused an important decrease in the CGC (Figure 2). These decreased CGC values when the H–US–C protocol was used corresponded to the immobilization of several hundreds of water molecules per gelator molecule more than the corresponding values when the H–C protocol was used; for example, **G1**, which uses S4 and S5 solutions, suffered CGC reductions of ca. 47 and 40%, respectively. The lowest values of CGC were obtained using S4 and PBS for both **G1** and **G2**. The decrease in CGC observed with the H–US–C protocol could be related to a greater efficiency in the formation of supramolecular interactions due to the impact of ultrasound on molecular reorganization [13,21].

### 2.4. Effect of Ultrasound on Gelation Kinetics

**Figure 3:**
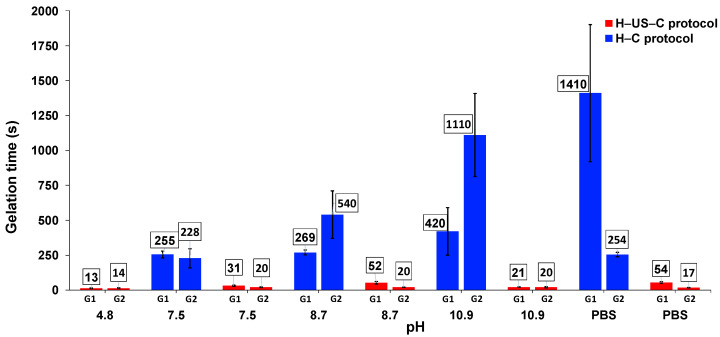
Comparison of the gelation times of **G1** and **G2** using the H–US–C protocol and H–C protocol at different pH values.

The gelation times were measured on the basis of the CGC values at the corresponding pH values, starting at the moment that heating was withdrawn. Interestingly, an enormous decrease in the gelation time was observed when the H–US–C protocol was used in comparison with the corresponding values when the H–C protocol was used (Figure 3). For example, we observed percentages that decreased between approximately 81% and 96% for **G1** and between 91% and 98% for **G2**.

Both the decrease in CGC and the drastic increase in the gelation rate with the H–US–C protocol highlight the ability of ultrasound to overcome the energetic and kinetic barriers associated with molecular self-assembly. This mechanism appears to be mediated by a cavitation phenomenon that accelerates primary nucleation processes and induces molecular restructuring and the kinetic trapping of high-energy supramolecular states, favoring the most effective intermolecular interactions while sacrificing early fibrillar nanotructures that are formed with defects during the self-assembly process [13,19,21].

### 2.5. Effect of Ultrasound on the Thermal Stability

**Figure 4:**
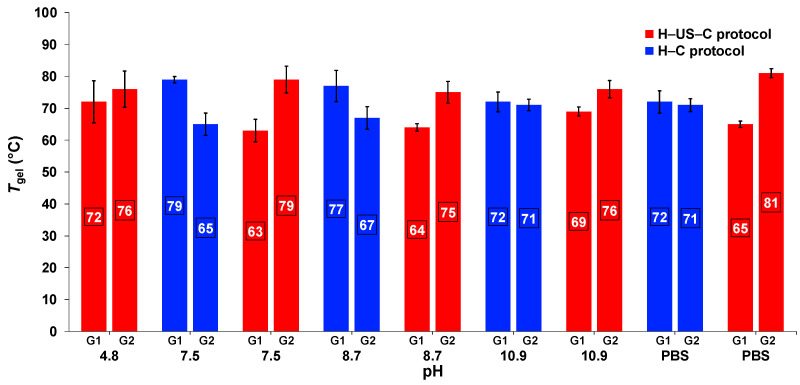
Comparison of the *T*_gel_ values of **G1** and **G2** obtained via the H–US–C protocol and H–C protocol at different pH values.

As shown in Figure 4, all *gel*-to-*sol* transition temperatures (*T*_gel_) ranged from 63 to 81 °C using the inverse flow method (see Section 4). The *T*_gel_ values obtained via the H–US–C protocol were, in most cases, similar to those obtained via the H–C protocol. However, for **G2**, a slight tendency for increased *T*_gel_ values of the hydrogels via the H–US–C protocol was observed, regardless of the pH value.

**Figure 5:**
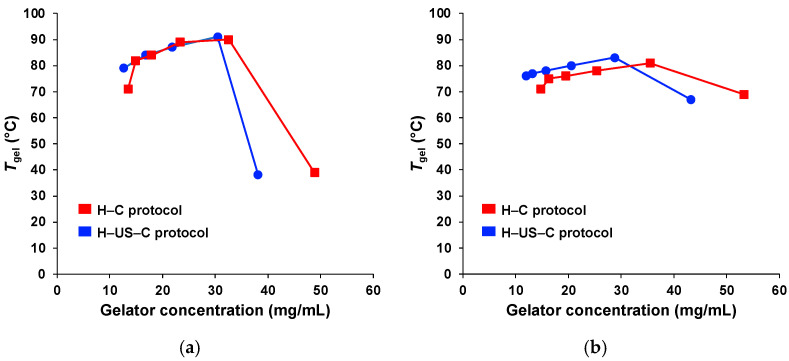
Evolution of *T*_gel_ as a function of gelator concentration of (**a**) **G1** and (**b**) **G2**, obtained using S6 solution.

As a typical feature of supramolecular gels, the *T*_gel_ increased considerably with an increasing initial gelator concentration, suggesting the formation of more closely packed networks, which emphasizes the impact on network density (Figure 5). For **G1**, Δ*T*_gel_ (calculated as the difference in *T*_gel_ values between the two preparation methods) values of 12 and 19 °C were observed for the H–US–C protocol and H–C protocol hydrogels, respectively. The maximum *T*_gel_ was ca. 90 °C; however, in both cases, the gelator concentration needed to obtain the maximum *T*_gel_ was lower when the H–US–C protocol was used. The concentration at the maximum *T*_gel_ was approximately three times greater than the corresponding value of CGC. Higher concentrations led to gel collapse. **G2** resulted in a lower Δ*T*_gel_ than **G1** did (Δ*T*_gel_ = 7 and 10 °C for the H–US–C protocol and H–C protocol, respectively). However, the *T*_gel_ values obtained at high concentrations (in the gel collapse region) were considerably greater for **G2** than for **G1**. As expected, the gelation kinetics became faster as the gelator concentration increased.

In terms of thermal responsiveness, all the hydrogels showed full thermoreversibility, with no significant changes in their *T*_gel_ values after several heating–cooling cycles. Moreover, the hydrogels were found to be stable at RT for at least two months without any visual changes in appearance and/or significant changes in *T*_gel._

### 2.6. Rheological Behavior

**Figure 6:**
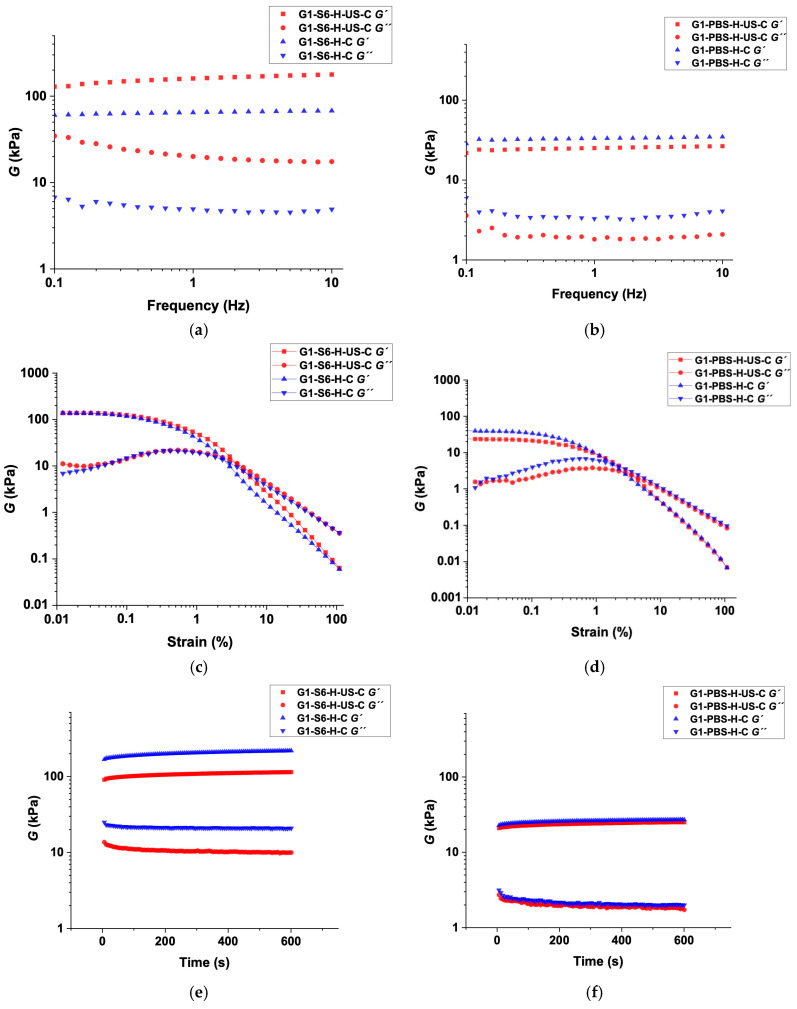
Oscillatory rheological measurements, DFS (**a**,**b**), DSS (**c**,**d**), and DTS (**e**,**f**), of native gel prepared using S6 and PBS solutions at the corresponding critical gelation concentrations of **G1** under the H–US–C and H–C protocols.

The viscoelastic nature of the hydrogels prepared via the H–C and H–US–C protocols was confirmed through oscillatory rheology for representative examples (Figure 6). For both gels prepared with solution S6 (pH = 10.9) and PBS (pH = 7.4), the linear viscoelastic regimes were defined by dynamic frequency sweep (DFS) (Figure 6a,b) and dynamic strain sweep (DSS) (Figure 6c,d) experiments. Within these regions, the storage modulus (*G′*) was found to be approximately one order of magnitude greater than the loss modulus (*G*′′) during the experiments, showing a low frequency dependence (i.e., **G1**-PBS-(H–US–C): G′ ≈ 23.8 kPa ± 1.02, G′′ ≈ 1.9 kPa ± 0.16; **G1**-PBS-(H–C): G′′ ≈ 25.9 kPa ± 0.96, G′′ ≈ 2.1 kPa ± 0.20; **G1**-S6-(H–US–C): G′ ≈ 107.4 kPa ± 5.52, G′′ ≈ 10.5 kPa ± 0.66; **G1**-S6-(H–C): G′ ≈ 202.7 kPa ± 12.41, G′′ ≈ 21.2 kPa ± 0.61). Importantly, the strain at which the systems collapsed improved. For gels made with the PBS solution via the H–C and H–US–C protocols, the strains used were 2% and 4%, respectively. For the gels made with the S6 solution via the H–C and H–US–C protocols, the strains were 2% and 5%, respectively. These observed increases in the strains at the breaking points highlight the enhancement of the ultrasound, resulting in more stable systems capable of withstanding greater external forces. Notably, in the case of the gels made with the S6 solution via the H–US–C protocol, they presented a higher *G′* modulus at a lower strain than the gels made via the H–C protocol, and, therefore, were better able to resist deformation processes.

On the other hand, dynamic time sweep (DTS) measurements at a 0.1% strain and a frequency of 1 Hz confirmed the stability of the systems over time at RT, as no phase changes were observed during the experiment (Figure 6e,f). The low tan δ values (also called the vibration damping coefficient) obtained for both protocols indicated that the systems behaved more like an elastic solid than a viscous fluid does, demonstrating a good tolerance against external forces (i.e., **G1**-S6-(H–US–C): tan δ ≈ 0.09 ± 0.01, **G1**-S6-(H–C): tan δ ≈ 0.10 ± 0.01, **G1**-PBS-(H–US–C): tan δ ≈ 0.08 ± 0.01, and **G1**-PBS-(H–C): tan δ ≈ 0.08 ± 0.01). In general, lower tan δ values are associated with a higher stiffness.

The similarity between the tan δ values from the H–C and H–US–C protocols indicates that both methods yielded gels with consistent mechanical properties. However, the H–US–C protocol not only decreased the CGC, but it also enhanced the gelation kinetics without compromising the thermal reversibility, homogeneity, and viscoelastic nature of the gels [13].

### 2.7. Morphological Characterization

**Figure 7:**
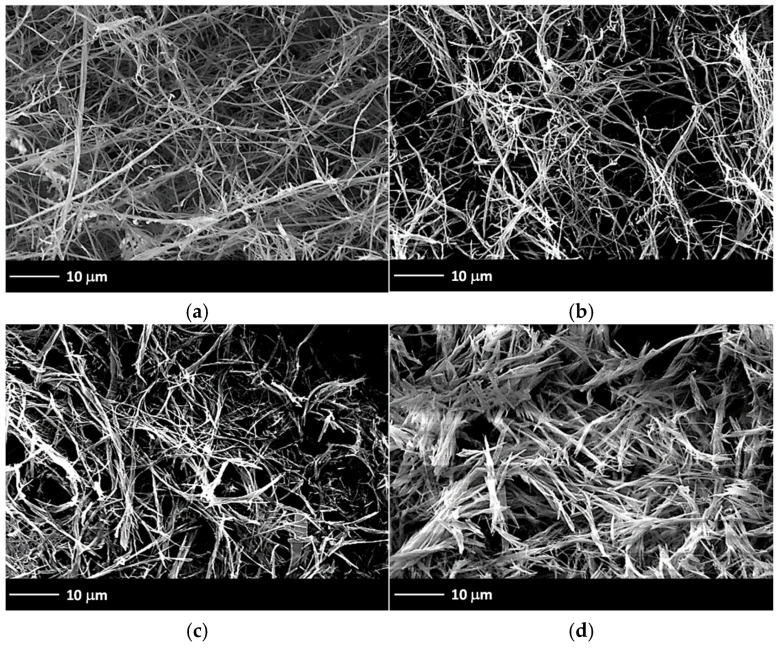
Representative SEM images of xerogels prepared by freeze-drying the corresponding hydrogels made of **G1** under different conditions: (**a**) PBS, *c* = 5.5 mg·L^−1^, H–C protocol; (**b**) PBS, *c* = 4.4 mg·L^−1^; H–US–C protocol; (**c**) S6, *c* = 13.5 mg·L^−1^, H–C protocol; and (**d**) S6, *c* = 12.7 mg·L^−1^, H–US–C protocol (magnification 1500×).

To gain a better understanding of the morphologies of the hydrogels, we performed scanning electron microscopy (SEM) on the corresponding xerogels. Generally, entangled fibrillar networks were observed for gels made from **G1**, regardless of the solvent or preparation method (Figure 7).

The samples prepared with PBS (pH = 7.4) presented large, longitudinal fibrillar structures (Figure 7a,b), whereas the hydrogels prepared with S6 solution (pH = 10.9) presented more densely packed structures with a spiky morphology (Figure 7c,d). These types of structures were consistently observed with an increasing gelator concentration, irrespective of the solvent. The smallest fibers for all cases showed diameters below 200 nm and lengths on the micron scale. Furthermore, no significant differences in hydrogel morphology were observed between the H–C and H–US–C preparation methods (see ESI, Figure S2, for more SEM images of the xerogels at different magnifications).

**Figure 8:**
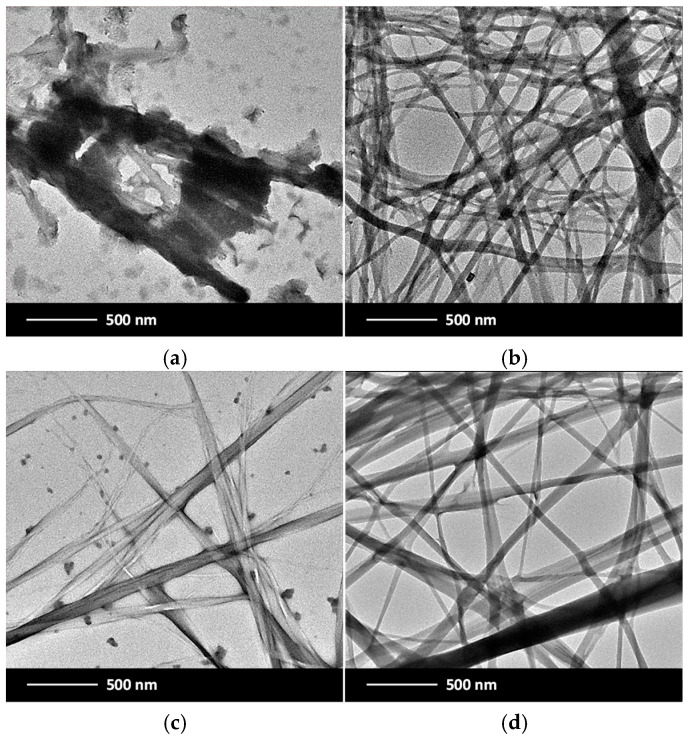
Representative TEM images of hydrogels made of **G1** under different conditions: (**a**) PBS, *c* = 5.5 mg·L^−1^, H–C protocol; (**b**) PBS, *c* = 4.4 mg·L^−1^; H–US–C protocol; (**c**) S6, *c* = 13.5 mg·L^−1^, H–C protocol; and (**d**) S6, *c* = 12.7 mg·L^−1^, H–US–C protocol (magnification 500 nm).

The previous observations were further supported by transmission electron microscopy (TEM). Hydrogels derived from **G1**, prepared via the H–C protocol, revealed a fibrillar network comprising bundles of twisted fibers with diameters ranging from approximately 30 to 80 nm (smallest feature) and lengths extending into the micron scale (Figure 8a,c). For the xerogels prepared via the H–US–C protocol, the fiber surfaces appeared notably smooth and uniform (Figure 8b,d). These samples also exhibited well-organized fibrils with widths between 30 and 80 nm and lengths exceeding 1 mm.

The high aspect ratio observed in the entangled networks can be attributed to anisotropic growth, which indicates that a well-ordered molecular arrangement formed nanofibers. The overall consistency between the SEM and TEM observations in our study confirms that the fibrillar morphology of the hydrogels was well preserved across different imaging techniques, reducing the likelihood of artifacts. However, it is essential to acknowledge that sample preparation for SEM and TEM imaging may induce significant microstructural changes. Consequently, these images must be interpreted with careful consideration (see ESI, Figure S3, for more TEM images).

### 2.8. Cytotoxicity

**Figure 9:**
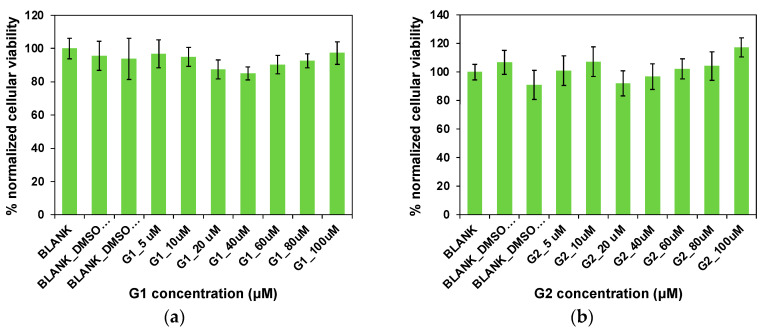
Cytotoxicity of (**a**) **G1** and (**b**) **G2** incubated in the presence of SH-SY5Y cells for 24 h at 37 °C. Untreated and cells incubated with 2 and 4 μL of dimethyl sulfoxide (DMSO) were used as controls. Cellular viability was calculated using the MTT assay. Error bars represent triplicates (SD = 3).

To assess the potential use of the designed small molecules **G1** and **G2**, cytotoxicity studies were conducted to evaluate their biocompatibility. **G1** and **G2** were incubated in the presence of SH-SY5Y cells as a neuronal cell model for 24 h at 37 °C in concentrations ranging from 5 to 100 µM. The cytotoxicity analysis was carried out using the well-known 3-(4,5-dimethylthiazol-2-yl)-2,5-diphenyltetrazolium bromide (MTT) colorimetric assay. As shown in Figure 9, none of the concentrations studied affected cell viability and no significant differences in cytotoxicity were found for either the **G1** or **G2** molecules. In this regard, the cellular viability rates were close to 100% in both cases, even at the highest concentrations of **G1** and **G2** (100 μM). These results may suggest the biocompatibility of the compounds **G1** and **G2**, as well as their potential for in vivo use.

### 2.9. In Vitro Drug Release

**Figure 10:**
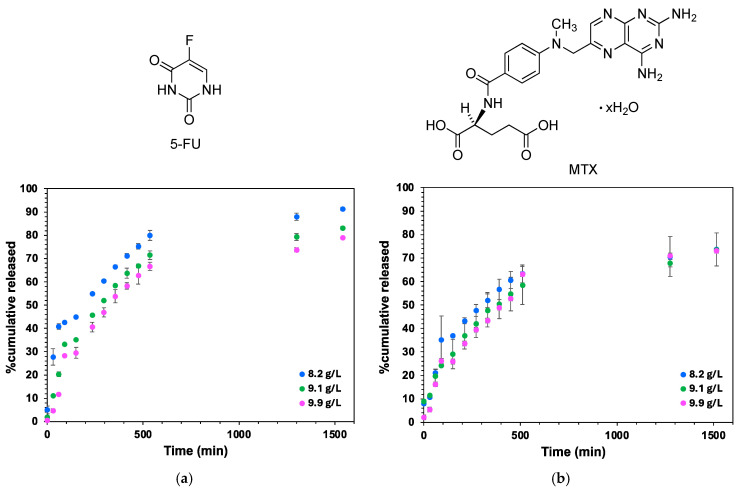
In vitro drug release behavior of (**a**) 5-FU and (**b**) MTX depending on the gelator concentration (8.2 g·L^−1^, blue spots; 9.1 g·L^−1^, green spots; and 9.9 g·L^−1^, pink spots). Hydrogels containing 5-FU and MTX were incubated in the presence of 1X PBS acting as a receptor phase at 37 °C. Data at different incubation times correspond to the average of three independent experiments (SD = 3). Chemical structures of 5-FU and MTX are included (*top*).

Prior to evaluating drug release experiments, the encapsulation efficiency (EE%) of two chemotherapeutic small molecules, namely 5-fluorouracil (5-FU) and methotrexate hydrate (MTX) (Figure 10), within a model hydrogel made of **G1** in PBS (pH 7.4) was estimated to be 83% and 81%, respectively (see Section 4). The ability of the model hydrogel made of **G1** to promote the release of 5-FU and MTX over time was investigated by varying different parameters such as the gelator concentration (8.2, 9.1, and 9.9 g·L^−1^) and pH (4.8 and 7.4). After initial experiments, the gelator concentrations above the CGC were chosen to ensure a greater stability of the gel when incorporating the drugs.

As expected, a small burst release of 5-FU and MTX was observed during the first incubation times and was directly correlated with the gelator concentration. In this regard, lesser amounts of 5-FU (4.7 ± 0.8%) and MTX (5.5 ± 1.8%) were released after the first 30 min incubation using a 9.9 g·L^−1^ gelator concentration. This behavior was tentatively justified by the increase in the internal entanglement of the gels, which would tend to limit the undesirable release of both drugs through the tridimensional matrices. Subsequently, the release behavior of 5-FU and MTX exhibited different cumulative trends after 26 h of incubation. In the case of 5-FU, a correlation between the amount of drug released (91.2 ± 0.8%, 83.1 ± 0.8%, and 79 ± 0.2%) and the gelator concentration (8.2, 9.1, and 9.9 g·L^−1^) was noticed, respectively, during the whole incubation process (Figure 10a).

However, in the case of MTX, slower and similar diffusion rates of the drug were observed with all concentrations tested (8.2, 9.1, and 9.9 g·L^−1^), resulting in 73.5 ± 0.5%, 73.6 ± 7.0%, and 73.2 ± 2.7% of MTX, respectively after 25 h of incubation (Figure 10b). This difference in the release behavior was mainly attributed to the nature of the entrapped molecule itself and its major tendency to interact with the fibrillar gel matrix via electrostatic interactions, hydrogen bonding, and π-π interactions. Therefore, it was hypothesized that these existing interactions might have affected the behavior of the drug, which contributed to the lower diffusion rate of MTX through the fibrillar gel matrix when compared to its 5-FU counterpart.

Finally, we also investigated how changes in the pH of the receptor phase affected the release of 5-FU and MTX. Note that engineering pH-responsive systems has enabled the construction of smart materials for cancer therapy capable of being injected directly at the tumor site, favoring the release of chemotherapeutic drugs, taking advantage of the slight acidic pH present in the tumor microenvironment [23,24]. To evaluate whether our gels were prone to responding to acid media and thus modulating the release of 5-FU and MTX, the following two pH values were considered: 4.8 and 7.4, but the gelator concentration was maintained constant (9.1 g·L^−1^). No significant changes in the release of both drugs were observed with an acidic pH (see ESI, Figure S18). In this regard, the release of 94 ± 2.4% of 5-FU was produced after 26 h of incubation when the pH of the receptor phase was 4.8. With respect to MTX, its cumulative release was slightly lower, as expected, though it reached values of 81.2 ± 0.6% in the same period of incubation time.

### 2.10. Drug Release Kinetics

**Figure 11:**
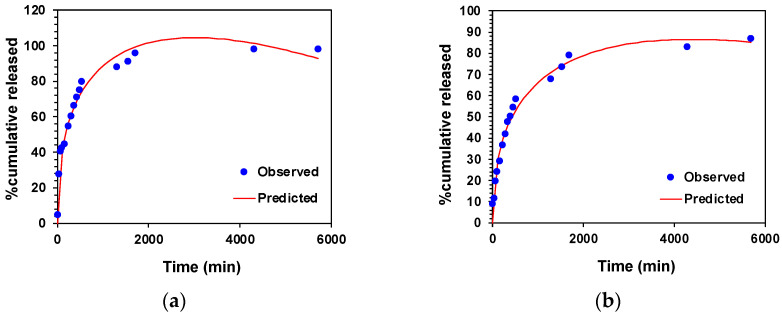
Fitting of (**a**) 5-FU and (**b**) MTX to Peppas–Sahlin equation model. Blue dots correspond to the experimental data (8.2 g·L^−1^ gelator concentration, PBS as a receptor phase, and pH 7.4). Red line corresponds with the predicted correlation to the experimental release.

The mechanism of 5-FU and MTX release from the hydrogels was studied by analyzing the experimental release data with respect to the first-order, Higuchi, Korsmeyer–Peppas, Peppas–Sahlin, and Weibull mathematical models. The parameters, constants, and correlation coefficients, including graphical representations of all these equation models, are displayed in the ESI (see Tables S3–S9 and Figures S19–S26). Interestingly, our results demonstrated that the Peppas–Sahlin model fit the experimental drug release data in accordance with the highest value of the correlation coefficient (R^2^), the lowest value of Akaike’s information criterion (AIC), and the largest model selection criterion (MSC) value in all the conditions studied, including the gelator concentration and pH. As an example, Figure 11 shows the fitting of (a) 5-FU and (b) MTX to the Peppas–Sahlin equation model using a gelator concentration of 8.2 g·L^−1^.

The Peppas–Sahlin equation model accounts for both the diffusion and relaxation mechanisms involved during the release of actives through composites. These two processes are determined by the two terms of the equation. In this sense, the first term of the equation (${K1}_{P-S}\times t^{m}$) refers the release of drugs by Fickian diffusion or Case I, whereas the second term (${K2}_{P-S}\times t^{2m})$ describes the Case II polymer chain relaxation contribution, which tends to be influenced by stress generated from swelling-controlled drug delivery composites [25,26]. To confirm which mechanism was predominant during the release of 5-FU and MTX, we observed that the diffusion constant K1 was significantly greater than the relaxation constant K2 in all cases (K1/K2 ratio > 1), as shown in Tables S3–S10 (ESI). This suggested that the diffusion mechanism predominated over the relaxation or erosion process during the release of 5-FU and MTX through the composites. Thus, if we take as an example the release data of 5-FU shown in Figure 11a, K1 provided a value of 5.69, K2 a value of −0.078, and a diffusion coefficient of *m* = 0.45 (Table S3). In the case of MTX (Figure 11b), the value of K1 was 3.97 and K2 was −0.046, with an *m* value of 0.45 (Table S6). Accordingly, this diffusion mechanism was also confirmed by the linear trend observed in a log–log plot of 5-FU and MTX release over time (see ESI, Figures S25 and S26 for 5-FU and MTX, respectively).

Additionally, the Weibull distribution model was also used to obtain insight into the mechanisms that governed the release of 5-FU and MTX. It should come as no surprise that this model also provided the best fitting equation for the release of the two drugs studied (R^2^ > 0.9), as this equation is fully empirical [27]. In this model, the mechanism of drug transport through composites is determined by a β exponent value. In this sense, the β value was found to be less than 0.75, also suggesting a Fickian diffusion mechanism in the release of both model drugs, as observed in the semi-empirical equation models listed above.

## 3. Conclusions and Outlook

In conclusion, the *N*-substituted Phe derivatives **G1** and **G2** are able to form a variety of biocompatible hydrogels in a wide range of pHs under both conventional heating–cooling (H–C) and heating–ultrasound–cooling protocols (H–US–C). The incorporation of ultrasound significantly improved the gelation kinetics and reduced the CGC of the hydrogels, highlighting its potential to optimize molecular self-assembly. These findings demonstrate the advantages of ultrasound as a non-invasive stimulus for creating adaptable and high-performing supramolecular gels. The results presented in this work are consistent with our previous studies, where ultrasound has been identified as a stimulus capable of inducing the self-repair of early-formed defective supramolecular networks, thus favoring gelation even in conditions where traditional methods such as H–C are not effective. Furthermore, the studied hydrogels exhibited a good encapsulation efficiency and tunable release profiles for 5-fluorouracil and methotrexate, driven by the gelator concentration and drug–matrix interactions, underscoring their suitability for controlled drug delivery applications.

It is important to emphasize that the application of ultrasound to induce the formation of supramolecular gels constitutes a powerful and versatile tool to improve properties such as CGC and gelation kinetics, improving at the same time, or at least without compromising, the reversibility and thermomechanical stability of such gels. These parameters are important, since they could be limiting factors in industrial processes, as well as in biomedical applications such as the development of matrices for drug release or tissue engineering.

The concept of kinetic trapping induced by ultrasonic cavitation, where supramolecular networks remain in higher energy states interrupting their natural evolution to lower energetic states, offers new opportunities for the design of adaptive and functional materials, which may even be unattainable or difficult to manufacture by conventional stimuli, such as heat or light. This phenomenon not only depends on the inherent molecular characteristics of the gelator, but also on the parameters of the ultrasonic device, such as the power, frequency, and duration of the treatment, which can be adjusted to modify the structural and functional properties of the material more precisely and in a controlled manner. We believe that advances in the fundamental understanding of the effect of ultrasound on molecular self-organization could pave the way towards the rational design of ultrasensitive gelators, establishing a solid foundation for future developments in the field using this technique.

## 4. Materials and Methods

### 4.1. Synthesis and Characterization of Compounds

#### 4.1.1. Materials

All reagents and solvents were purchased from commercial suppliers and used without further purification. For aldehydes, a distillation process was necessary to maintain their purity during the investigation.

#### 4.1.2. Characterization Methods

Thin-layer chromatography (TLC) was performed on 0.25 mm silica gel 60-F_254_ plates provided by Merck (Darmstadt, Germany), and ninhydrin and/or phosphomolybdic acid was used for stains.

^1^H NMR spectra were recorded at 500 MHz, and ^13^C NMR-APT spectra were recorded at 125 MHz via a Bruker Avance-500 (Research Support General Service (SEGAI), University of La Laguna). Chemical shifts are denoted in δ (ppm) relative to residual solvent peaks. The coupling constants, *J*, are given in Hertz. The following standard abbreviations were used for the characterization of the ^1^H NMR signals: s = singlet, d = doublet, t = triplet, m = multiplet, dd = doublet of doublets, dt = doublet of triplets. In all cases, a solution of D_2_O with KOH (0.4 M) was used as the deuterated solvent.

FTIR-ATR spectra were recorded via a Cary 360 FTIR-ATR spectrophotometer in transmittance mode. The spectra were recorded via 150 scans between 650 and 4000 cm^−1^ at a 2 cm^−1^ spectral resolution.

All the melting points were determined by using a STUART SMP10 melting point apparatus by the open capillary tube method and were uncorrected.

Elemental analyses were carried out by using a CNHS Flash EA 1112 instrument (SEGAI, Universidad de La Laguna).

#### 4.1.3. Synthetic Procedures

The general synthetic procedures are given below. Spectroscopic and other characterization data are provided in the Supporting Information (see ESI, Figures S4–S17).

- General procedure for the synthesis of compounds **1**, **2**, **4**, **5**, **6**, and **7**

Phenylalanine derivatives were synthesized starting from the ester of the amino acid and by the means of the following two steps: reductive amination and hydrolysis.

Reductive amination was performed following the procedure described by Qiao and coworkers [28], with slight modifications. In all cases, TEA (1 equiv) and liquid aldehyde (1.2 equiv) were added to a solution of amino acid methyl ester hydrochloride salt (1 equiv) in methanol (0.2 M). The reaction mixture was stirred at RT for 4 h to promote the formation of the imine, after which it was treated with NaBH_4_ (2 equiv) at 0 °C to obtain the corresponding amine. After 8 h, the solvent was removed, and the crude product was dissolved in ethyl acetate, washed with brine (3 times), dried over anhydrous MgSO_4_, concentrated under vacuum, and used in the next step without further purification.

Afterwards, basic hydrolysis was conducted by dissolving the ester (1 equiv) and KOH (2 equiv) in a methanol–water mixture (*v*/*v* 1:1, 72 mM). This mixture was stirred and refluxed for 5 h. Once the reaction mixture was cooled to RT, the pH was adjusted to ca. 6–7 using HCl (1 M) and NaHCO_3_ (1.2 M)_._ The resulting mixture was stirred for 30 min until the product precipitated. The solvent was removed by filtration, and the solid was washed several times with deionized water, suspended in DCM, filtered again, and dried under vacuum.

- General procedure for the synthesis of compound **3**

Compound **3** was prepared via the procedure proposed by Mandal and co-workers [29], with slight modifications. Briefly, benzaldehyde (1 equiv) and NaOH (2 equiv) were added to a methanol–water mixture (*v*/*v* 1:1, 0.2 M) containing L-Phe. The mixture was refluxed for 18 h and then cooled to RT. NaBH_4_ (2 equiv) was added, and the mixture was stirred for 12 h. The pH was adjusted via glacial acetic acid, and the mixture was stirred for 30 min until the product precipitated. The solvent was removed by filtration, and the solid was washed several times with deionized water, suspended in DCM, and dried under vacuum.

### 4.2. Preparation and Characterization of Hydrogels

#### 4.2.1. Preparation of Hydrogels

The weighed phenylalanine-based gelator was placed inside a screw-capped glass vial (3.2 cm height and 1 cm diameter), and 0.5 mL of aqueous solution was added. The mixture was gently heated with a heat gun until the solid was completely dissolved. Two treatments were subsequently studied, (1) the normal H–C protocol and (2) the H–US–C protocol involving the ultrasound treatment at 120 W, 40 kHz (ultrasound bath JP-Selecta^TM^ 3000865), and a constant temperature (25 ± 2 °C) directly after heating until gel formation.

To evaluate the dependence of pH on gelation ability, preliminary screening was performed using Universal Britton–Robinson buffer with pH values ranging from 1.93 to 13.74 (solutions S1–S7) (see ESI, Tables S1 and S2). In addition, the effect of PBS (0.01 M, pH 7.4) was also tested.

#### 4.2.2. Physicochemical Characterization of Hydrogels

The CGC was determined via the addition of 5 mg of gelator, and the aqueous solution was added to small portions (starting with 50 µL) until it was dissolved. In all cases, the initial concentration for the gelation tests was 100 g·L^−1^. The concentration at which the solution exhibited no gravitational flow upon the inversion of the vial was considered as the CGC.

The *T*_gel_ values were determined visually via the inverse flow method, as previously described [30]. The sealed vial was loaded into a thermoblock, and the temperature was raised at a rate of 2 °C·min^−1^. The gel stability was confirmed every 2 min, and the *T*_gel_ was defined as the temperature at which the hydrogel melted and the solvent, under the influence of gravity, started to flow out of the gel structure when the vial was inverted. The average values of at least two random experiments were given. We previously validated the inverse flow method with other physical gels by correlation of the obtained *T*_gel_ value with the first endothermic peak observed by DSC [13,21].

Rheological measurements were performed at 25 °C with an AR 2000 Advanced rheometer (TA Instruments) equipped with a Julabo C cooling system and a plane–plate geometry (40 mm in diameter, 1000 µm gap size). In all cases, 3 mL of gel was prepared inside a screw-capped glass vial (5.5 cm in height and 2.5 cm in diameter) and left undisturbed for at least 24 h before the measurement. The following three experiments were carried out for each sample, as previously described [20]: (a) DSS was performed by increasing the amplitude of deformation from 0.1 to 100% at 1 Hz. (b) DFS between 0.1 and 100 Hz at a 0.1% strain. (c) DTS using the initial strain (0.1%) at 1 Hz for 10 min.

The morphological characterization of the bulk samples was carried out by SEM and transmission electron microscopy TEM (SEGAI, University of La Laguna). (a) SEM: images were obtained via a ZEISS EVO 15 emission scanning electron microscope (SEM, resolution 2 nm). Sample preparation: Xerogel samples were prepared via freeze-drying. Prior to imaging, a 5 nm Au film was sputtered (40 mA, 30 s) on the samples placed on carbon tape. (b) TEM: images were recorded via a JEOL JEM 1010 TEM microscope with a resolution of 0.4 nm. For sample preparation, 10 mL of the gel suspension was allowed to adsorb onto carbon-coated grids (300 mesh, from Aname, Madrid, Spain). The grid was placed over a piece of filter paper to absorb the excess solvent.

### 4.3. Cytotoxicity Assays

#### 4.3.1. Materials

For cell culture experiments, 1 L of RNase-free water was treated overnight with 1.0 mL of DEPC and then autoclaved to ensure sterility. PBS tablets, sourced from Merck Sigma-Aldrich (Saint Louis, MO, USA), were dissolved in DEPC-treated water to prepare a 0.01 M PBS solution. Dubelcco’s Modified Eagle Medium (DMEM) was purchased from Capricorn Scientific (Ebsdorfergrund, Germany) and supplemented with 10% Fetal Bovine Serum (FBS) (Thermofisher Scientific, Waltham, MA, USA). Cytotoxicity studies utilized MTT from Merck Sigma-Aldrich (St. Louis, MO, USA) and DMSO from Panreac Química (Barcelona, Spain).

#### 4.3.2. Experimental Protocol

To sustain exponential growth, SH-SY5Y cells, which were provided by the Cryogenics core facility of the University of Barcelona, were routinely passaged. One day before the experiment, the cells were seeded in a 96-well plate at a density of 15,000 cells per well, using complete DMEM with 10% fetal bovine serum. After 24 h of incubation, solutions of **G1** and **G2** were prepared in DMSO at a concentration of 1 mg·mL^−1^. A determined working concentration volume of both compounds was added to each well to reach final concentrations of 5, 10, 20, 40, 60, 80, and 100 µM in a volume of 200 µL, respectively. Samples were incubated with SH-SY5Y cells for 24 h at 37 °C. After the incubation time, the culture medium was removed and replaced with fresh DMEM (200 µL). The cells were incubated for an additional 15 h, after which, 20 µL of an MTT solution (5 mg·mL^−1^) was added to each well. The final mixture was incubated for 3 h at 37 °C. Finally, the culture medium was removed, and formazan crystals were dissolved in 200 µL of DMSO. Cell viability was calculated by analyzing the final absorbance at 570 nm using a Biotek Synergy H1 Hybrid Multi-Mode Reader (Agilent Technologie, Santa Clara, CA, USA). Untreated cells and cells incubated with 2 μL and 4 μL of DMSO were used as controls. Experiments were carried out in triplicate and normalized cellular viability was calculated using Equation (1).

(1) $$normalized\;cellular\;viability=\frac{Absorbance\;treated\;SH-SY5Y\;cells}{Absorbance\;control\;cells}\times 100$$

### 4.4. In Vitro Drug Release

#### 4.4.1. Experimental Procedure

Drug encapsulation was carried out using 5-fluorouracil (5-FU) and methotrexate hydrate (MTX) as model drugs to determine the release behavior of low- and high-soluble water-soluble small-molecule drugs, respectively. Briefly, the gelator was loaded in a screw-capped glass vial (3.2 cm height and 1 cm diameter) and then dissolved in 0.5 mL of PBS at 80 °C. Then, 0.5 mL of drug in PBS was mixed with the hydrogel obtained after cooling for a few minutes, and the final solution was homogenized by gently heating to 50 °C. The resulting solution was left undisturbed at H–C for 12 h to achieve a drug-entrapped self-supported hydrogel. The EE was calculated by UV spectrophotometry (5-FU: λ_max_ = 266 nm; MTX: λ_max_ = 302 nm) through the standard calibration measurement and applying Equation (2).

(2) $$EE=\frac{m_{i}-m_{t=0}}{m_{i}}$$

where $m_{i}$ represents the initial amount of drug encapsulated and $m_{t=0}$ represents the amount of drug in the release media at t = 0.

In vitro control release experiments were performed using 1 mL of receptor media, and the gel was maintained at 37 °C. At specific time intervals, aliquots of 250 µL from the release media were removed and replaced with fresh solution. After centrifugation at 10,000 rpm for 10 min, the supernatant of the release media was collected and stored at −20 °C until analysis. The concentration of the drug in the collected supernatants was determined via UV–visible spectrophotometry (Genesys 180 spectrophotometer, Thermo Scientific, Waltham, MA, USA) with reference to a calibration curve. The cumulative release of the drug was calculated according to Equation (3) [31].

(3) $$Q=C_{n}V_{t}+V_{s}\sum C_{n-1}$$

where Q represents the cumulative released weight, $C_{n}$ represents the drug concentration at time t, $V_{t}$ represents the volume of medium ($V_{t}$ = 1000 µL), and $V_{s}$ represents the volume of the solution removed from the supernatant ($V_{t}$ = 250 µL).

The in vitro drug release data were fitted to the first-order, Higuchi, Korsmeyer–Peppas, Peppas–Sahlin, and Weibull models via Equations (4)–(8), respectively. In this context, M∞ and Mt represent the maximum and cumulative amounts of active agent (5-FU or MTX) released over time, respectively. Constants such as K_F-O_, K_H_, K_K–P_, K_P–S_, and K_WB_ provide insights into the structural and geometric characteristics of the dosage form and *n* or *m* are the diffusional exponents. When diffusional exponents are approximately 0.5, a Fickian mechanism (Case I) is considered to be predominant. For values of 0.5 < n < 1 (Case II), the diffusion is classified as non-Fickian. Concerning the Weibull equation, α is a constant and β characterizes different diffusion mechanisms, including (i) Fickian for β ≤ 0.75 and (ii) complex processes that incorporate diffusion mechanisms for 0.75 < β < 1.

The DDSolver add-in program [32] was employed to fit both the FU and MTX release data to the aforementioned models.

(4) $$\frac{M_{t}}{M_{\infty }}=100\times (1-e^{-K_{F-O}\times t})$$

(5) $$\frac{M_{t}}{M_{\infty }}=K_{H}\times \sqrt{t}$$

(6) $$\frac{M_{t}}{M_{\infty }}=K_{K-P}\times t^{n}$$

(7) $$\frac{M_{t}}{M_{\infty }}={K1}_{P-S}\times t^{m}+{K2}_{P-S}\times t^{2m}$$

(8) $$\frac{M_{t}}{M_{\infty }}=\alpha \times (1-{e^{-(K_{WB}\times t)}}^{\beta })$$

#### 4.4.2. Statistical Analysis and Mathematical Modeling

Statistical analysis was used, and the values were statistically compared via one-way Analysis of Variance (ANOVA) (SPSS Statistics). In all cases, post hoc comparisons of the means of individual groups (via ANOVA) were performed via Tukey’s honestly significant difference test. A significance level of *p* < 0.05 was used to denote significance in all the cases.

## Data Availability

The original contributions presented in this study are included in the article/Supplementary Materials. Further inquiries can be directed to the corresponding authors.

## Supplementary Materials

The following supporting information can be downloaded at: www.mdpi.com/article/10.3390/gels11030160/s1, Figure S1: Optical appearance of hydrogels; Figure S2: (A–H) Representative SEM images of xerogels prepared by freeze-drying the corresponding hydrogels made of **G1** under different conditions: (A) and (B) PBS, *c* = mg·L^−1^, H–C protocol; (C) and (D) PBS, *c* = 4.4 mg·L^−1^, H–US–C protocol; (E) and (F) S6, *c* = 13.5 mg·L^−1^, H–C protocol; (G) and (H) S6, *c* = 12.7 mg·L^−1^, H–US–C protocol (A, C, E, and G, magnification 500× and B, D, F, and H, magnification 2000KX); Figure S3: (A–H) Representative TEM images of hydrogels made of **G1** under different conditions: (A) and (B) PBS, *c* = 5.5 mg·L^−1^, H–C; protocol; (C) and (D) PBS, *c* = 4.4 mg·L^−1^, H–US–C protocol; (E) and (F) S6, *c* = 13.5 mg·L^−1^, H–C protocol; (G) and (H) S6, *c* = 12.7 mg·L^−1^, H–US–C protocol; Figure S4: ^1^H NMR (500 MHz, D_2_O-KOH) of (furan-2-ylmethyl)-L-phenylalanine (**1**); Figure S5: ^13^C NMR (125 MHz, D_2_O-KOH) of (furan-2-ylmethyl)-L-phenylalanine (**1**); Figure S6: ^1^H NMR (500 MHz, D_2_O-KOH) of (thiophen-2-ylmethyl)-L-phenylalanine (**2**); Figure S7: ^13^C NMR (125 MHz, D_2_O-KOH) of (thiophen-2-ylmethyl)-L-phenylalanine (**2**); Figure S8: ^1^H NMR (500 MHz, D_2_O-KOH) of benzyl-L-phenylalanine (**3**); Figure S9: ^13^C NMR (125 MHz, D_2_O-KOH) of benzyl-L-phenylalanine (**3**); Figure S10: ^1^H NMR (500 MHz, D_2_O-KOH) of (pyridin-2-ylmethyl)-L-phenylalanine (**4**); Figure S11: ^13^C NMR (125 MHz, D_2_O-KOH) of (pyridin-2-ylmethyl)-L-phenylalanine (**4**); Figure S12: ^1^H NMR (500 MHz, D_2_O-KOH) of (pyridin-4-ylmethyl)-L-phenylalanine (**5**); Figure S13: ^13^C NMR (125 MHz, D_2_O-KOH) of (pyridin-4-ylmethyl)-L-phenylalanine (**5**); Figure S14: ^1^H NMR (500 MHz, D_2_O-KOH) of (4-(pyridin-4-yl)benzyl)-L-phenylalanine (**6**); Figure S15: ^13^C NMR (125 MHz, D_2_O-KOH) of (4-(pyridin-4-yl)benzyl)-L-phenylalanine (**6**); Figure S16: ^1^H NMR (500 MHz, D_2_O-KOH) of isopentyl-L-phenylalanine (**7**); Figure S17: ^13^C NMR (125 MHz, D_2_O-KOH) of isopentyl-L-phenylalanine (**7**); Figure S18: In vitro drug release of 5-FU (A) and MTX (B) as a function of pH (4.8) keeping the gelator concentration constant (9.1 g·L^−1^). Hydrogel was incubated at 37 °C. Aliquots were withdrawn and the same volume was added to the receptor phase. Error bars correspond to the measurement of three replicates (SD = 3); Figure S19: Fitting of 5-FU to First-order (A), Higuchi (B), Korsmeyer–Peppas (C), Peppas–Sahlin (D), and Weibull (E). Blue dots correspond to the experimental data (8.2 g·L^−1^ gelator concentration, PBS as a receptor phase, and pH 7.45). The DDSolver add-in program was used to fit the release data of 5-FU to the models described above. Blue dots correspond to the experimental data and red line corresponds with the predicted correlation to the experimental release; Figure S20: Fitting of 5-FU to First-order (A), Higuchi (B), Korsmeyer–Peppas (C), Peppas–Sahlin (D), and Weibull (E). Blue dots correspond to the experimental data (9.1 g·L^−1^ gelator concentration, PBS as a receptor phase, and pH 7.45). The DDSolver add-in program was used to fit the release data of 5-FU to the models described above. Blue dots correspond to the experimental data and red line corresponds with the predicted correlation to the experimental release; Figure S21: Fitting of 5-FU to First-order (A), Higuchi (B), Korsmeyer–Peppas (C), Peppas–Sahlin (D), and Weibull (E). Blue dots correspond to the experimental data (9.9 g·L^−1^ gelator concentration, PBS as a receptor phase, and pH 7.45). The DDSolver add-in program was used to fit the release data of 5-FU to the models described above. Blue dots correspond to the experimental data and red line corresponds with the predicted correlation to the experimental release; Figure S22: Fitting of MTX to First-order (A), Higuchi (B), Korsmeyer–Peppas (C), Peppas–Sahlin (D), and Weibull (E). Blue dots correspond to the experimental data (8.2 g·L^−1^ gelator concentration, PBS as a receptor phase, and pH 7.45). The DDSolver add-in program was used to fit the release data of MTX to the models described above. Blue dots correspond to the experimental data and red line corresponds with the predicted correlation to the experimental release; Figure S23: Fitting of MTX to First-order (A), Higuchi (B), Korsmeyer–Peppas (C), Peppas–Sahlin (D), and Weibull (E). Blue dots correspond to the experimental data (9.1 g·L^−1^ gelator concentration, PBS as a receptor phase, and pH 7.45). The DDSolver add-in program was used to fit the release data of MTX to the models described above. Blue dots correspond to the experimental data and red line corresponds with the predicted correlation to the experimental release; Figure S24: Fitting of MTX to First-order (A), Higuchi (B), Korsmeyer–Peppas (C), Peppas–Sahlin (D), and Weibull (E). Blue dots correspond to the experimental data (9.9 g·L^−1^ gelator concentration, PBS as a receptor phase, and pH 7.45). The DDSolver add-in program was used to fit the release data of MTX to the models described above. Blue dots correspond to the experimental data and red line corresponds with the predicted correlation to the experimental release; Figure S25: Log-log plot of 5-FU released versus time varying the gelator concentration (A) 8.2 g·L^−1^; (B) 9.1 g·L^−1^; and (C) 9.9 g·L^−1^. The first 60% of drug released was considered in this study; Figure S26: Log-log plot of MTX released versus time varying the gelator concentration (A) 8.2 g·L^−1^; (B) 9.1 g·L^−1^; and (C) 9.9 g·L^−1^. The first 60% of drug released was considered in this study; Table S1: Composition of the Britton–Robinson Buffer; Table S2: Buffer solutions titrated with NaOH (0.2 M). Table S3: Constant values, correlation coefficient, AIC, and MSC values obtained from equation models studied for the release of 5-FU varying the gelator concentration (8.2 g·L^−1^) and pH (7.45); Table S4: Constant values, correlation coefficient, AIC, and MSC values obtained from equation models studied for the release of 5-FU varying the gelator concentration (9.1 g·L^−1^) and pH (7.45); Table S5: Constant values, correlation coefficient, AIC, and MSC values obtained from equation models studied for the release of 5-FU varying the gelator concentration (9.9 g·L^−1^) and pH (7.45); Table S6: Constant values, correlation coefficient, AIC, and MSC values obtained from equation models studied for the release of MTX varying the gelator concentration (8.2 g·L^−1^) and pH (7.45); Table S7: Constant values, correlation coefficient, AIC, and MSC values obtained from equation models studied for the release of MTX varying the gelator concentration (9.1 g·L^−1^) and pH (7.45); Table S8: Constant values, correlation coefficient, AIC, and MSC values obtained from equation models studied for the release of MTX varying the gelator concentration (9.9 g·L^−1^) and pH (7.45); Table S9: Constant values, correlation coefficient, AIC, and MSC values obtained from equation models studied for the release of 5-FU keeping the gelator concentration constant (9.1 g·L^−1^) and varying the pH (4.8); Table S10: Constant values, correlation coefficient, AIC, and MSC values obtained from equation models studied for the release of MTX keeping the gelator concentration constant (9.1 g·L^−1^) and varying the pH (4.8). Refs. [33,34] are cited in Supplementary Materials.

## Abbreviations

The following abbreviations are used in this manuscript:

AIC	Akaike’s information criterion
ANOVA	Analysis of variance
CGC	Critical gelation concentration
DCM	Dichloromethane
DFS	Dynamic frequency sweep
DMEM	Dubelcco’s modified eagle medium
DMSO	Dimethyl sulfoxide
DSC	Differential scanning calorimetry
DSS	Dynamic strain sweep
DTS	Dynamic time sweep
EE	Encapsulation efficiency
FBS	Fetal bovine serum
FTIR-ATR	Fourier transform infrared spectroscopy-attenuated total reflectance
H–C	Heating–cooling
H–US–C	Heating–ultrasound–cooling
5-FU	5-Fluorouracil
MSC	Model selection criterion
MTT	3-(4,5-Dimethylthiazol-2-yl)-2,5-diphenyltetrazolium bromide
NMR	Nuclear magnetic resonance
PBS	Phosphate-buffered saline
Phe	Phenylalanine
RT	Room temperature
SEM	Scanning electron microscopy
TEA	Triethylamine
TEM	Transmission electron microscopy
*T*_gel_	*Gel*-to-*sol* transition temperature
UV	Ultraviolet
