# Supplementary material for: Ultrasound-Enhanced Gelation of Stimuli-Responsive and Biocompatible Phenylalanine-Derived Hydrogels
Source: Gels. 2025 Feb 23;11(3):160. doi: 10.3390/gels11030160 (PMC11942152; doi:10.3390/gels11030160)
Supplement: Supplementary file 1 [file gels-11-00160-s001.zip › gels-3482181-supplementary.pdf]

SUPPORTING INFORMATION

Ultrasound-Enhanced Gelation of Stimuli-Responsive and Biocompatible Phenylalanine-Derived Hydrogels

Eduardo Buxaderas [1,2], Yanina Moglie [1,2,3], Aarón Baz Figueroa [2,3], Juan V. Alegre-Requena [4], Santiago Grijalvo [5], César Saldías [6], Raquel P. Herrera [7], Eugenia Marqués-López [7]* and David Díaz Díaz [2,3,8]*

1 Instituto de Química del Sur, INQUISUR (CONICET-UNS), Departamento de Química, Universidad Nacional del Sur, Av. Alem 1253, 8000 Bahía Blanca, Argentina; ebuxaderas@gmail.com (E.B.), yamoglie@ull.edu.es (Y.M.).

2 Instituto Universitario de Bio-Orgánica Antonio González, Universidad de La Laguna, Avda. Astrofísico Francisco Sánchez 2, La Laguna 38206, Tenerife, Spain; yamoglie@ull.edu.es (Y.M.), alu0100897234@ull.edu.es (A.B.F.), ddiazdiaz@ull.edu.es (D.D.D.)

3 Departamento de Química Orgánica, Universidad de La Laguna, Avda. Astrofísico Francisco Sánchez 3, La Laguna 38206, Tenerife, Spain; ebuxaderas@gmail.com (E.B.), yamoglie@ull.edu.es (Y.M.), alu0100897234@ull.edu.es (A.B.F.), ddiazdiaz@ull.edu.es (D.D.D.)

4 Departamento de Química Inorgánica, Instituto de Síntesis Química y Catálisis Homogénea (CSIC-University of Zaragoza), C/ Pedro Cerbuna 12, 50009 Zaragoza, Spain; jv.alegre@csic.es (J.V.A.-R.).

5 CIBER-BBN, ISCII. Jordi Girona 18-26, 08034 Barcelona, Spain; sgrgma@cid.csic.es (S.G.).

6 Departamento de Química Física, Facultad de Química y de Farmacia, Pontifica Universidad Católica de Chile; casaldia@uc.cl (C.S.).

7 Departamento de Química Orgánica, Laboratorio de Organocatálisis Asimétrica, Instituto de Síntesis Química y Catálisis Homogénea (CSIC-University of Zaragoza), C/ Pedro Cerbuna 12, 50009 Zaragoza, Spain; raquelph@unizar.es (R.P.H.), mmaamarq@unizar.es (E.M.-L.).

8 Institute of Organic Chemistry, University of Regensburg, Universitätsstr. 31, Regensburg 93040, Germany, Germany; ddiazdiaz@ur.de (D.D.D.).

* Correspondence: mmaamarq@unizar.es (E.M.-L.); ddiazdiaz@ull.edu.es (D.D.D.)

TABLE OF CONTENTS

1. Optical appearance of hydrogels 3

2. Preparation of aqueous solutions 4

3. Additional SEM and TEM images 5

4. Characterization data of *N*-substituted phenylalanine derivatives 6

5. NMR spectra of *N*-substituted phenylalanine derivatives 8

6. Drug delivery study 15

1. Optical appearance of hydrogels

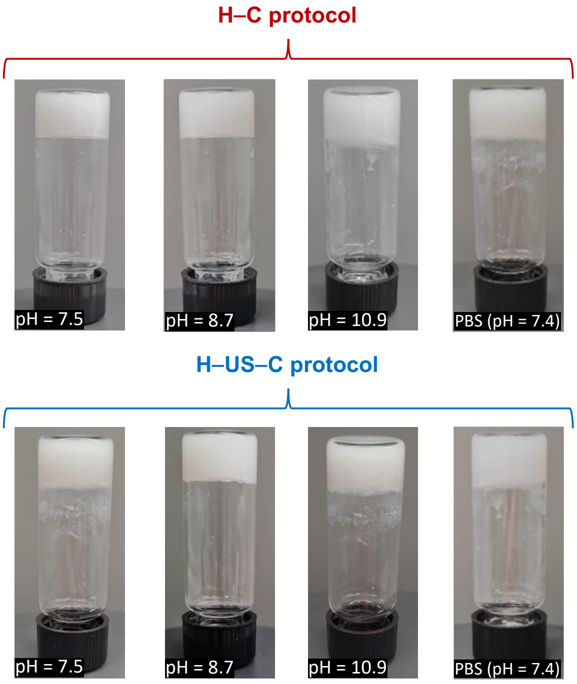

Figure S1. Optical appearance of representative hydrogels obtained from **G1**.

2. Preparation of aqueous solutions

Table S1. Composition of the Britton-Robinson buffer.

**Compound**	**Initial concentration (stock solution) (M)**	**Amount for 500 mL**	**Concentration in solution (M)**
AcOH	17.5	1.15 mL	0.04
H_3PO_4	17.2	1.16 mL	0.04
H_3BO_3	--	1.24 g	0.04

Britton-Robinson (BR) aqueous universal buffer solutions pH = 1.93–13.74 were prepared by mixing appropriate volumes of acids and basic buffer components. The acidic buffer component includes 0.04 M acetic acid, 0.04 M phosphoric acid, and 0.04 M boric acid. The basic buffer component is 0.2 M sodium hydroxide solution.

Table S2. Buffer solutions titrated with NaOH (0.2 M).

**Solution**	**pH**	**Buffer volume (mL)**	**NaOH (mL)**
1	1.93 ± 0.13	10	0
2	3.08 ± 0.12	10	1.50
3	4.84 ± 0.14	10	2.80
4	7.48 ± 0.15	10	5.00
5	8.67 ± 0.14	10	6.10
6	10.87 ± 0.30	10	7.55
7	13.74 ± 0.21	0	10

The pH was measured with a Hanna pH-meter (HI 991001, equipped with HI 1296D pH/temperature electrode). The pH meter was calibrated with standard buffer solutions with pH buffers 4, 7, and 10 before each use.

3. Additional SEM and TEM images

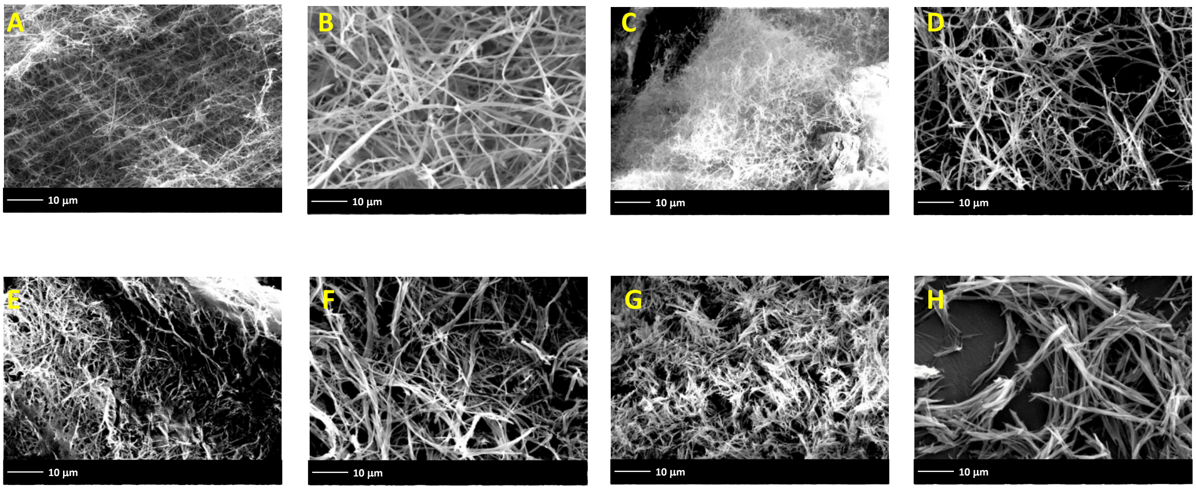

Figure S2. Representative SEM images of xerogels prepared by freeze-drying the corresponding hydrogels made of **G1** under different conditions: (A) and (B) PBS, *c* = 5.5 mgL^{-1}, H–C protocol; (C) and (D) PBS, *c* = 4.4 mgL^{-1}, H–US–C protocol; (E) and (F) S6, *c* = 13.5 mgL^{-1}, H–C protocol; (G) and (H) S6, *c* = 12.7 mgL^{-1}, H–US–C protocol (A, C, E and G, magnification 500x and B, D, F, and H, magnification 2000KX).

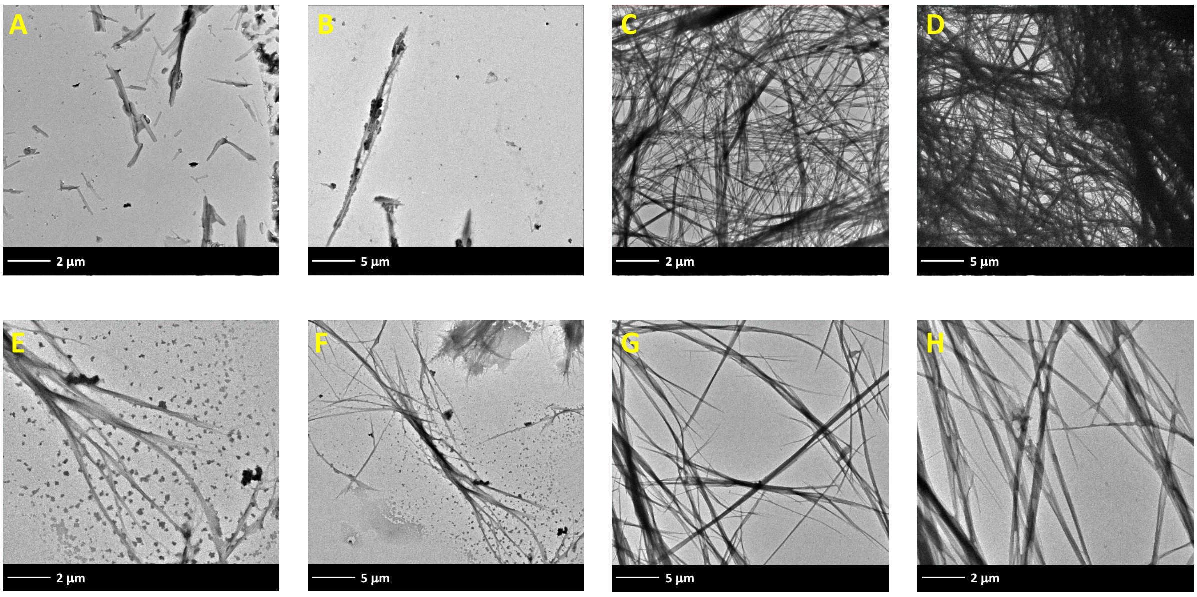

Figure S3. Representative TEM images of hydrogels made of **G1** under different conditions: (A) and (B) PBS, *c* = 5.5 mgL^{-1}, H–C; protocol; (C) and (D) PBS, *c* = 4.4 mgL^{-1}, H–US–C protocol; (E) and (F) S6, *c* = 13.5 mgL^{-1}, H–C protocol; (G) and (H) S6, *c* = 12.7 mgL^{-1}, H–US–C protocol.

4. Characterization data of *N*-substituted phenylalanine derivatives

(Furan-2-ylmethyl)-L-phenylalanine (1): White solid. Mp 185–196 °C Decomp. ^{1}H NMR (500 MHz, D_2O-KOH) δ 7.33 (d, *J* = 1.89 Hz, 1H), 7.21 (t, *J* = 7.28 Hz, 2H), 7.18–7.13 (m, 1H), 7.11–7.08 (m, 2H), 6.27 (dd, *J* = 3.21, 1.84 Hz, 1H), 6.12 (d, *J* = 3.16 Hz, 1H), 3.61 (d, *J* = 14.13 Hz, 1H), 3.47 (d, *J* = 14.22 Hz, 1H), 3.19 (dd, *J* = 7.39, 6.23 Hz, 1H), 2.80–2.69 (m, 2H). ^{13}C NMR (125 MHz, D_2O-KOH) δ 180.9, 152.6, 142.6, 138.1, 129.3, 128.6, 126.6, 110.4, 107.9, 64.1, 43.4, 39.1. IR (cm^{-1}) ν 3086, 1602, 1498, 1431, 1379, 1330, 1287, 1185, 1148, 1080, 1017, 980, 918, 864, 820, 749. Elemental analysis calcd for $C_{14}H_{15}NO_3$: C 68.56; H 6.16; N 5.71; O 19.57, found C 68.19; H 5.99; N 5.56; O 20.26.

(Thiophen-2-ylmethyl)-L-phenylalanine (2) [1]: White solid. Mp 190–202 °C Decomp. ^{1}H NMR (500 MHz, D_2O-KOH) δ 7.30–7.26 (m, 3H), 7.19 (dd, *J* = 8.56, 6.46 Hz, 1H), 7.16–7.12 (m, 2H), 6.93 (dd, *J* = 5.14, 3.39 Hz, 1H), 6.89 (d, *J* = 3.45 Hz, 1H), 3.85 (d, *J* = 13.88 Hz, 1H), 3.72 (d, *J* = 13.95 Hz, 1H), 3.29 (t, *J* = 6.91 Hz, 1H), 2.86–2.75 (m, 2H). ^{13}C NMR (125 MHz, D_2O-KOH) δ 180.9, 141.8, 138.1, 129.3, 128.6, 127.1, 126.6, 126.6, 125.4, 63.9, 45.3, 39.1. IR (cm^{-1}) ν 3028, 1607, 1435, 1390, 1330, 1272, 1205, 1159, 1081, 1028, 971, 916, 868, 794, 749, 697.

Benzyl-L-phenylalanine (3) [2]: White solid. Mp 185–208 °C Decomp. ^{1}H NMR (500 MHz, D_2O-KOH) δ 7.26–7.17 (m, 5H), 7.16–7.11 (m, 3H), 7.07 (dd, *J* = 7.01, 1.82 Hz, 2H), 3.58 (d, *J* = 12.8 Hz, 1H), 3.41 (d, *J* = 12.79 Hz, 1H), 3.19 (dd, *J* = 7.61, 6.22 Hz, 1H), 2.74 (qd, *J* = 13.49, 6.91 Hz, 2H). ^{13}C NMR (125 MHz, D_2O-KOH) δ 181.1, 139.0, 138.3, 129.5, 129.3, 128.7, 128.6, 128.6, 127.4, 126.6, 126.6, 64.5, 51.0, 39.1. IR (cm^{-1}) ν 3034, 1599, 1494, 1431, 1394, 1334, 1259, 1207, 1084, 1028, 980, 924, 868, 806, 745, 693.

(Pyridin-2-ylmethyl)-L-phenylalanine (4) [3]: White solid. Mp 184–200 °C Decomp. ^{1}H NMR (500 MHz, D_2O-KOH) δ 8.32 (dt, *J* = 4.84, 1.34 Hz, 1H), 7.70 (td, *J* = 7.73, 1.81 Hz, 1H), 7.27 – 7.17 (m, 5H), 7.15 (dd, *J* = 6.96, 1.73 Hz, 2H), 3.75 (d, *J* = 13.82 Hz, 1H), 3.58 (d, *J* = 13.83 Hz, 1H), 3.27 (t, *J* = 6.91 Hz, 1H), 2.83 (d, *J* = 6.89 Hz, 2H). ^{13}C NMR (125 MHz, D_2O-KOH) δ 180.9, 157.8, 148.4, 138.2, 138.1, 129.3, 128.6, 126.6, 123.0, 122.9, 64.8, 52.2, 39.2. IR (cm^{-1}) ν 3063, 3030, 2322, 1654, 1589, 1498, 1435, 1379, 1297, 1200, 1110, 1043, 1017, 872, 842, 767, 693, 667.

(Pyridin-3-ylmethyl)-L-phenylalanine (5) [3]: White solid. Mp 199–228 °C Decomp. ^{1}H NMR (500 MHz, D_2O-KOH) δ 8.31 (dd, *J* = 4.94, 1.63 Hz, 1H), 8.26 (d, *J* = 2.18 Hz, 1H), 7.62 (dt, *J* = 7.87, 1.92 Hz, 1H), 7.29 (dd, *J* = 7.88, 4.93 Hz, 1H), 7.24 (t, *J* = 7.25 Hz, 2H), 7.21 – 7.15 (m, 1H), 7.14 – 7.09 (m, 2H), 3.67 (d, *J* = 13.11 Hz, 1H), 3.49 (d, *J* = 13.31 Hz, 1H), 3.22 (t, *J* = 6.92 Hz, 1H), 2.80 (d, *J* = 6.92 Hz, 2H). ^{13}C NMR (125 MHz, D_2O-KOH) δ 180.9, 148.7, 147.5, 138.1, 137.7, 134.9, 129.3, 128.6, 126.6, 124.2, 64.5, 48.4, 39.1. IR (cm^{-1}) ν 2571, 1606, 1528, 1457, 1379, 1334, 1297, 1196, 1084, 998, 913, 831, 741, 697.

(4-(Pyridin-4-yl)benzyl)-L-phenylalanine (6): White solid. Mp 200–254 °C Decomp. ^{1}H NMR (500 MHz, D_2O-KOH) δ 8.25 (d, *J* = 5.37 Hz, 1H), 7.32 (d, *J* = 7.66 Hz, 2H), 7.28 (d, *J* = 5.25 Hz, 2H),

7.20–7.04 (m, 7H), 3.60 (d, *J* = 13.06 Hz, 1H), 3.41 (d, *J* = 13.06 Hz, 1H), 3.21 (t, *J* = 6.85 Hz, 1H), 2.83–2.71 (m, 2H). ^{13}C NMR (125 MHz, D_2O-KOH) δ 181.0, 149.0, 148.3, 140.4, 138.3, 135.7, 129.3, 129.2, 128.5, 126.9, 126.5, 121.6, 64.4, 50.7, 39.1. IR (cm^{-1}) ν 3030, 1599, 1490, 1435, 1379, 1338, 1297, 1203, 1129, 1080, 1032, 995, 916, 864, 808, 745, 700. Elemental analysis calcd for $C_{21}H_{20}N_2O_2$: C 75.88; H 6.06; N 8.43; O 9.63, found C 75.57; H 5.93; N 8.15; C 10.35.

Isopentyl-L-phenylalanine (7): White solid. Mp 205–236 °C Decomp. ^{1}H NMR (500 MHz, D_2O-KOH) δ 7.28 (t, *J* = 7.42 Hz, 2H), 7.24–7.17 (m, 3H), 3.22 (dd, *J* = 8.09, 5.73 Hz, 1H), 2.84 (dd, *J* = 13.32, 5.70 Hz, 1H), 2.75 (dd, *J* = 13.37, 8.13 Hz, 1H), 2.49–2.41 (m, 1H), 2.38–2.31 (m, 1H), 1.47 (dt, *J* = 13.38, 6.67 Hz, 1H), 1.25 (ddt, *J* = 8.61, 6.64, 3.23 Hz, 2H), 0.77 (t, *J* = 6.65 Hz, 6H). ^{13}C NMR (125 MHz, D_2O-KOH) δ 181.5, 138.3, 129.4, 129.3, 128.6, 126.6, 65.3, 45.4, 39.1, 37.8, 25.6, 22.2, 21.7. IR (cm^{-1}) ν 3037, 2963, 2870, 1565, 1453, 1375, 1300, 1177, 1058, 1025, 972, 894, 842, 771, 745, 700. Elemental analysis calcd for $C_{14}H_{21}NO_2$: C 71.46; H 9.00; N 5.95; O 13.60, found C 71.16; H 8.77; N 5.83; O 14.24.

5. NMR spectra of *N*-substituted phenylalanine derivatives

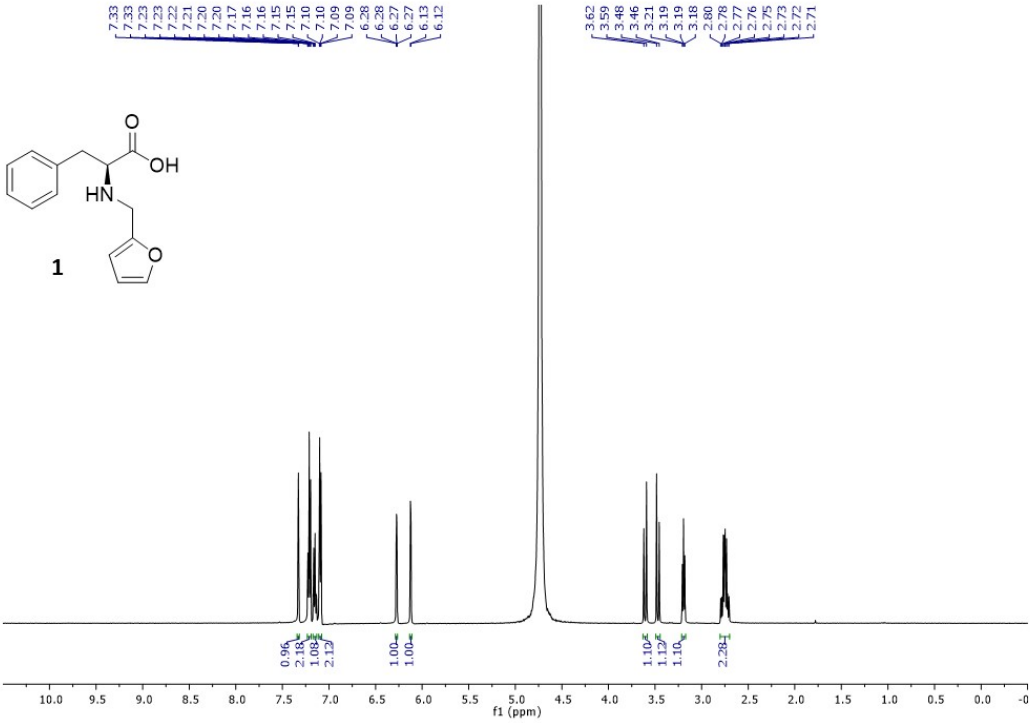

Figure S4. ^{1}H NMR (500 MHz, D_2O-KOH) of (furan-2-ylmethyl)-L-phenylalanine (**1**).

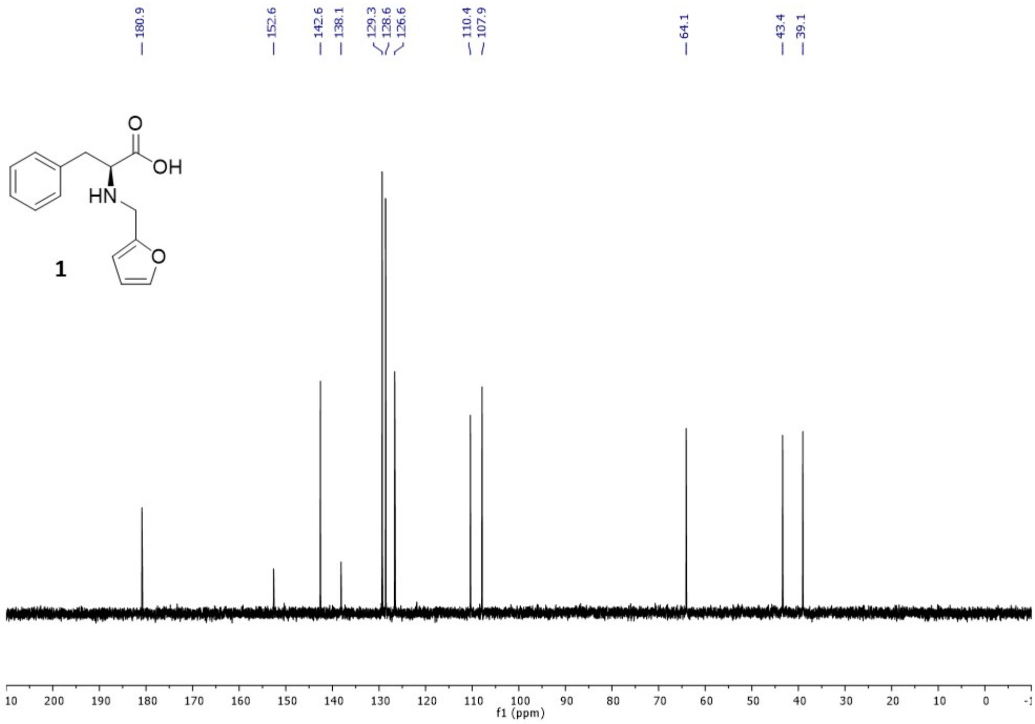

Figure S5. ^{13}C NMR (125 MHz, D_2O-KOH) of (furan-2-ylmethyl)-L-phenylalanine (**1**).

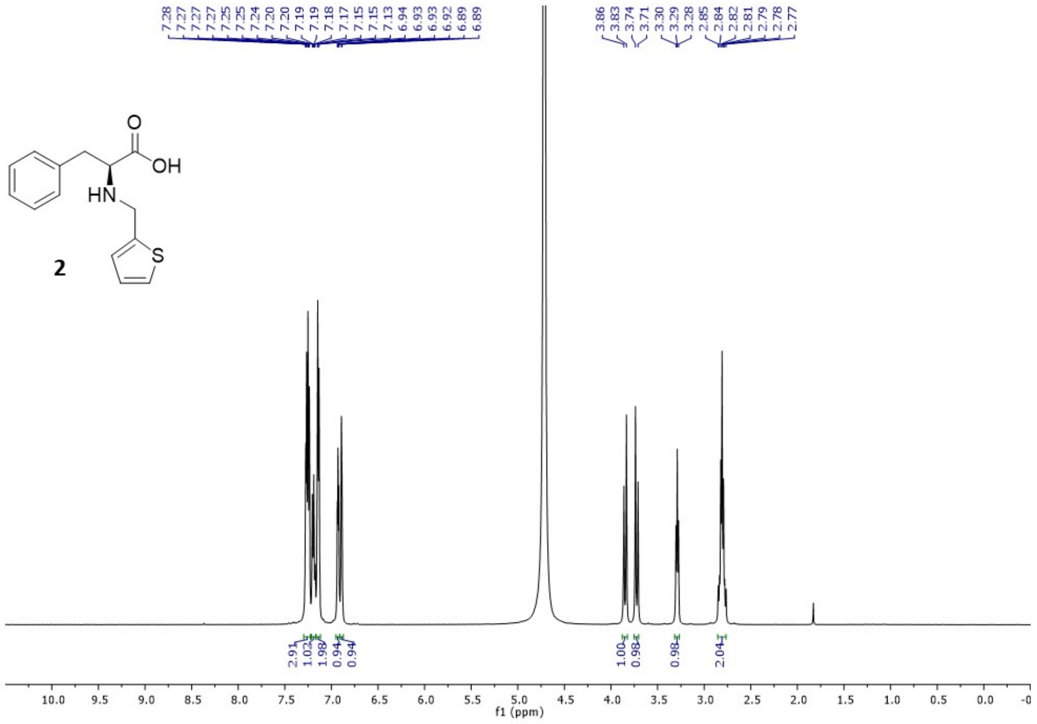

Figure S6. ^{1}H NMR (500 MHz, D_2O-KOH) of (thiophen-2-ylmethyl)-L-phenylalanine (**2**).

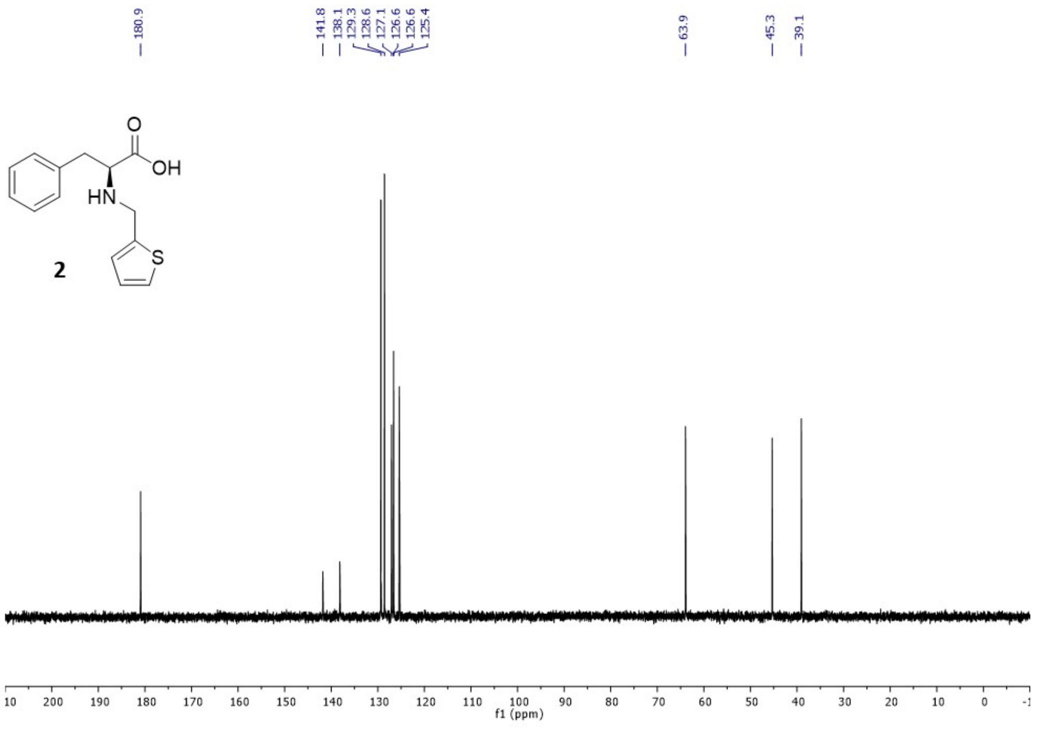

Figure S7. ^{13}C NMR (125 MHz, D_2O-KOH) of (thiophen-2-ylmethyl)-L-phenylalanine (**2**).

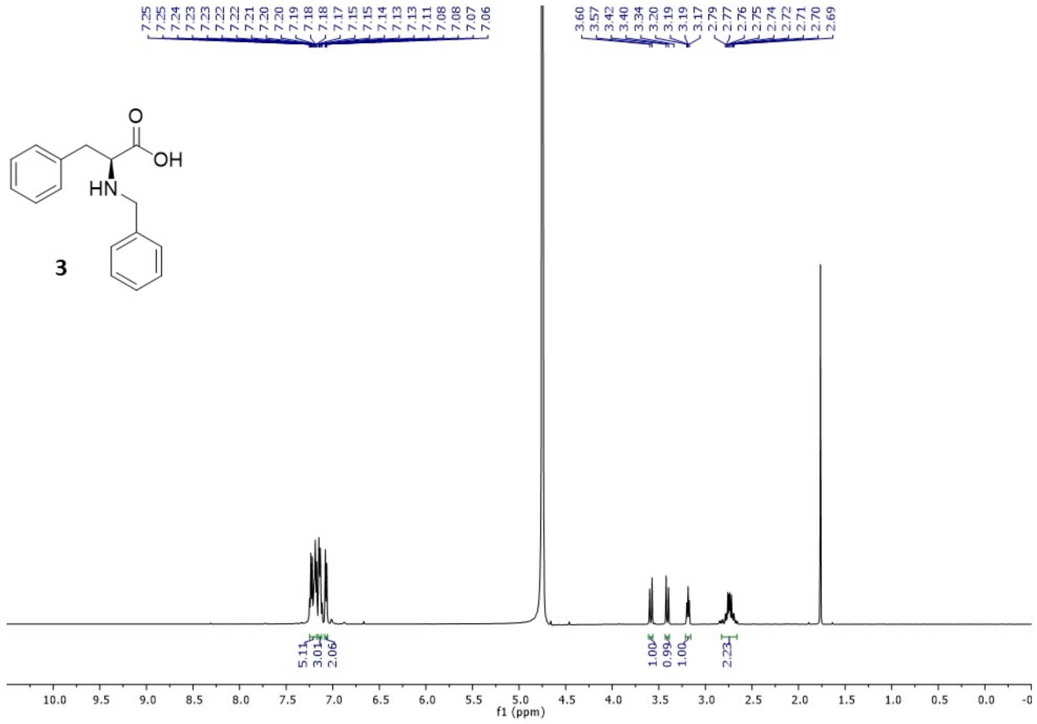

Figure S8. ^{1}H NMR (500 MHz, D_2O-KOH) of benzyl-L-phenylalanine (**3**).

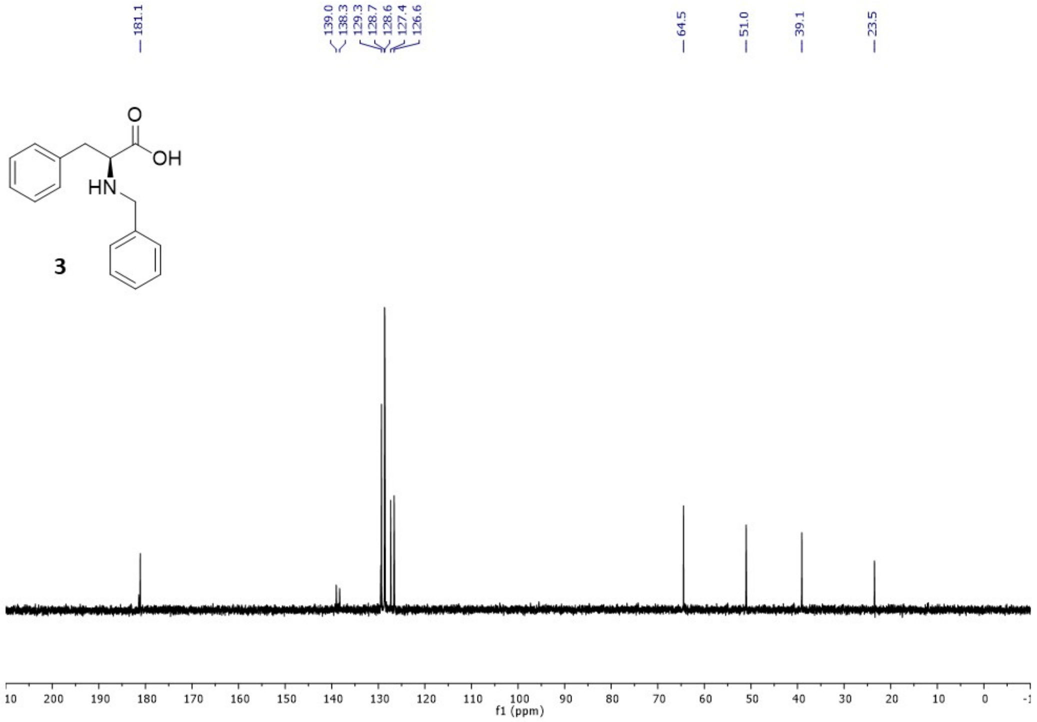

Figure S9. ^{13}C NMR (125 MHz, D_2O-KOH) of benzyl-L-phenylalanine (**3**).

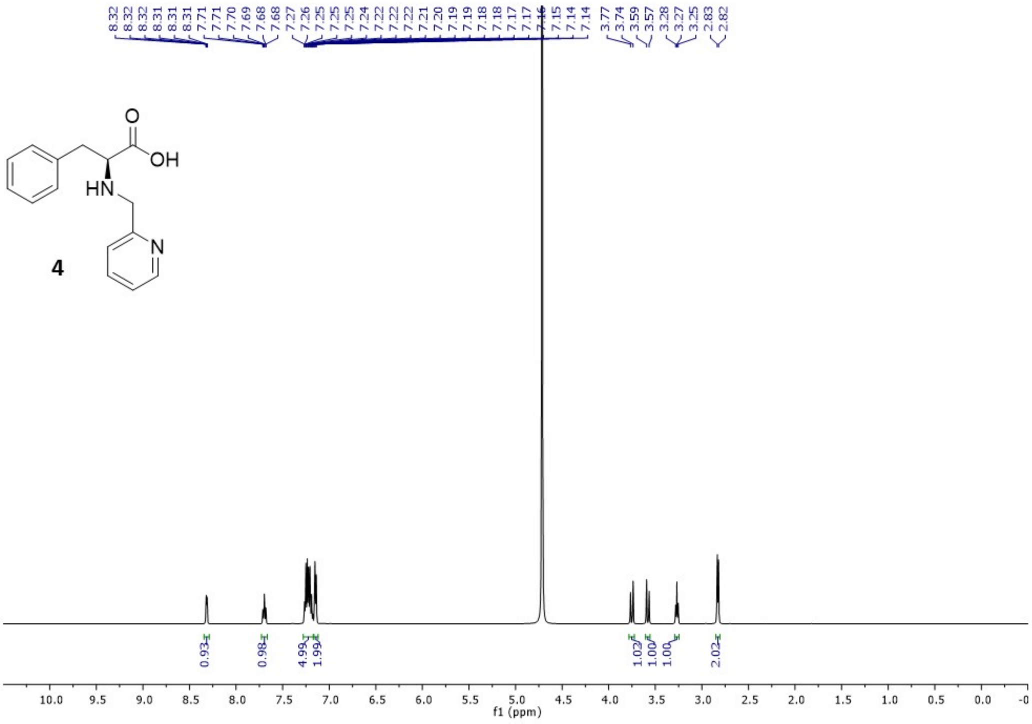

Figure S10. ^{1}H NMR (500 MHz, D_2O-KOH) of (pyridin-2-ylmethyl)-L-phenylalanine (**4**).

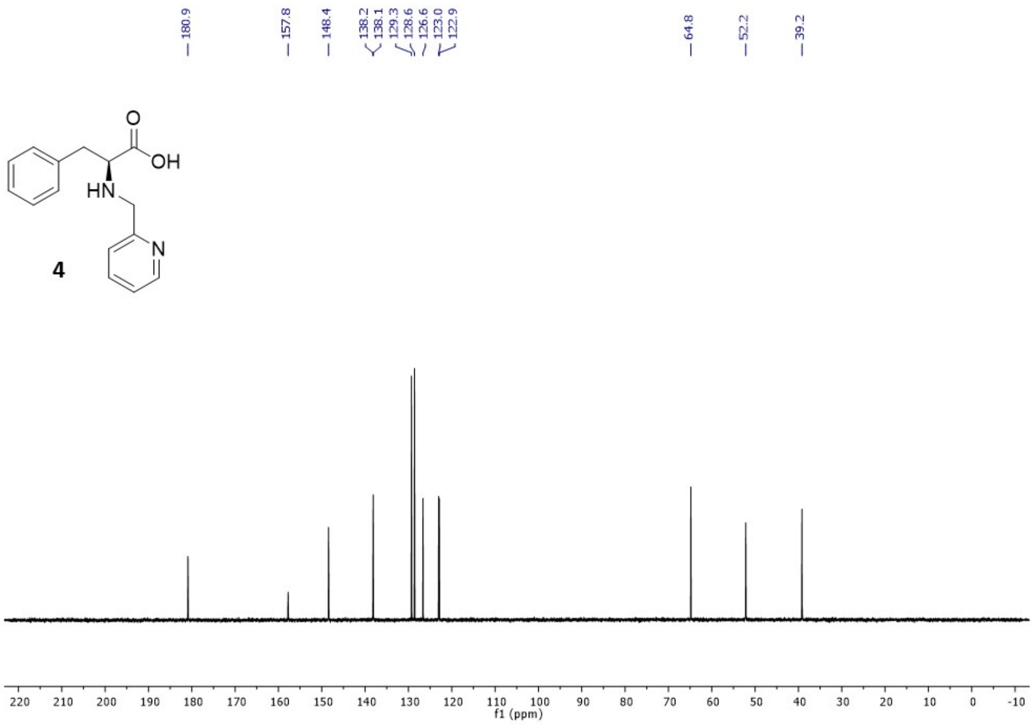

Figure S11. ^{13}C NMR (125 MHz, D_2O-KOH) of (pyridin-2-ylmethyl)-L-phenylalanine (**4**).

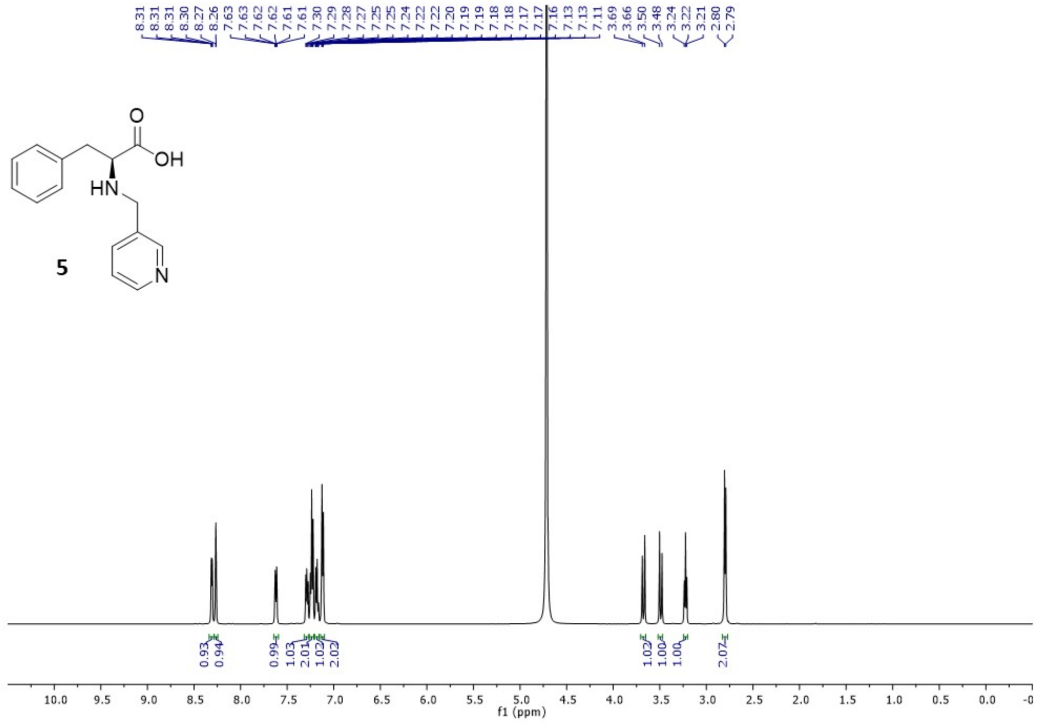

Figure S12. ^{1}H NMR (500 MHz, D_2O-KOH) of (pyridin-4-ylmethyl)-L-phenylalanine (**5**).

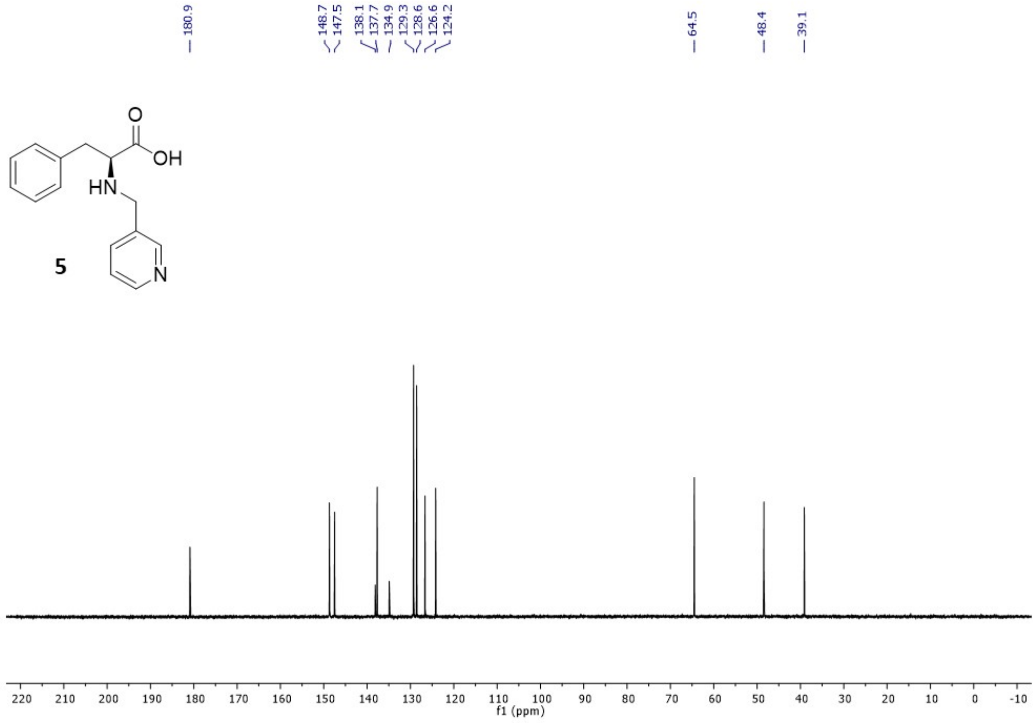

Figure S13. ^{13}C NMR (125 MHz, D_2O-KOH) of (pyridin-4-ylmethyl)-L-phenylalanine (**5**).

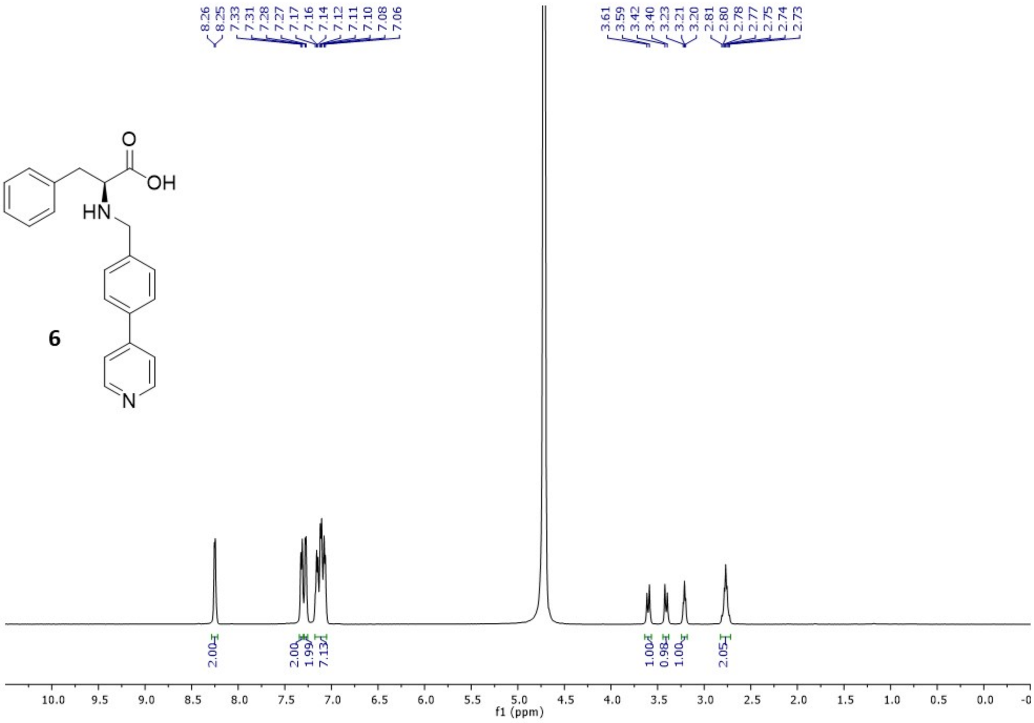

Figure S14. ^{1}H NMR (500 MHz, D_2O-KOH) of (4-(pyridin-4-yl)benzyl)-L-phenylalanine (**6**).

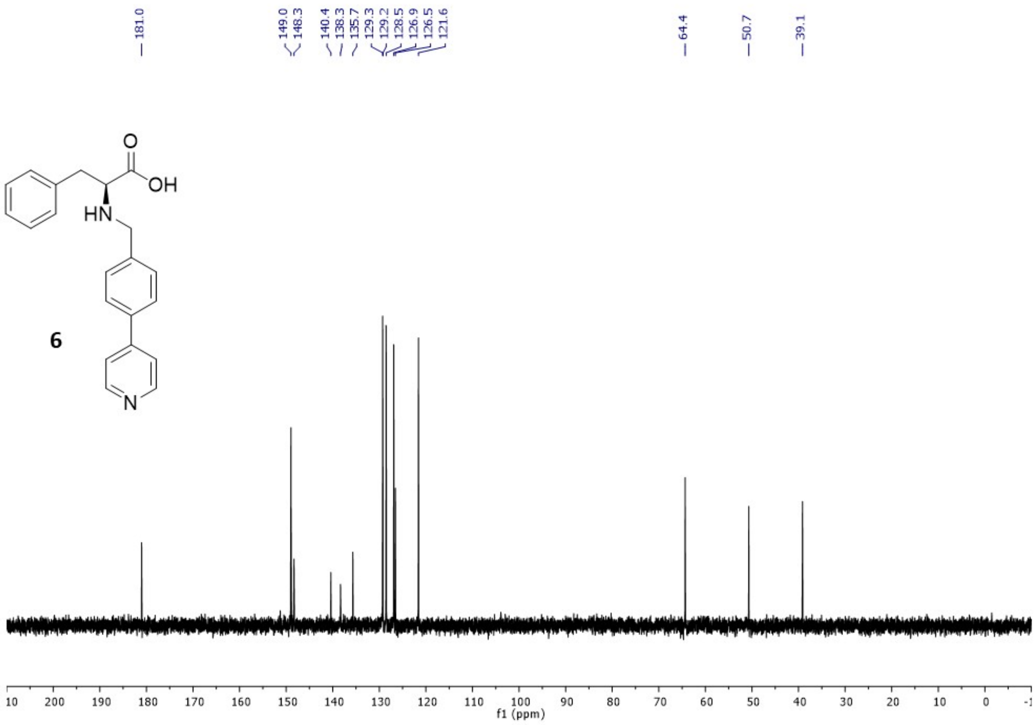

Figure S15. ^{13}C NMR (125 MHz, D_2O-KOH) of (4-(pyridin-4-yl)benzyl)-L-phenylalanine (**6**).

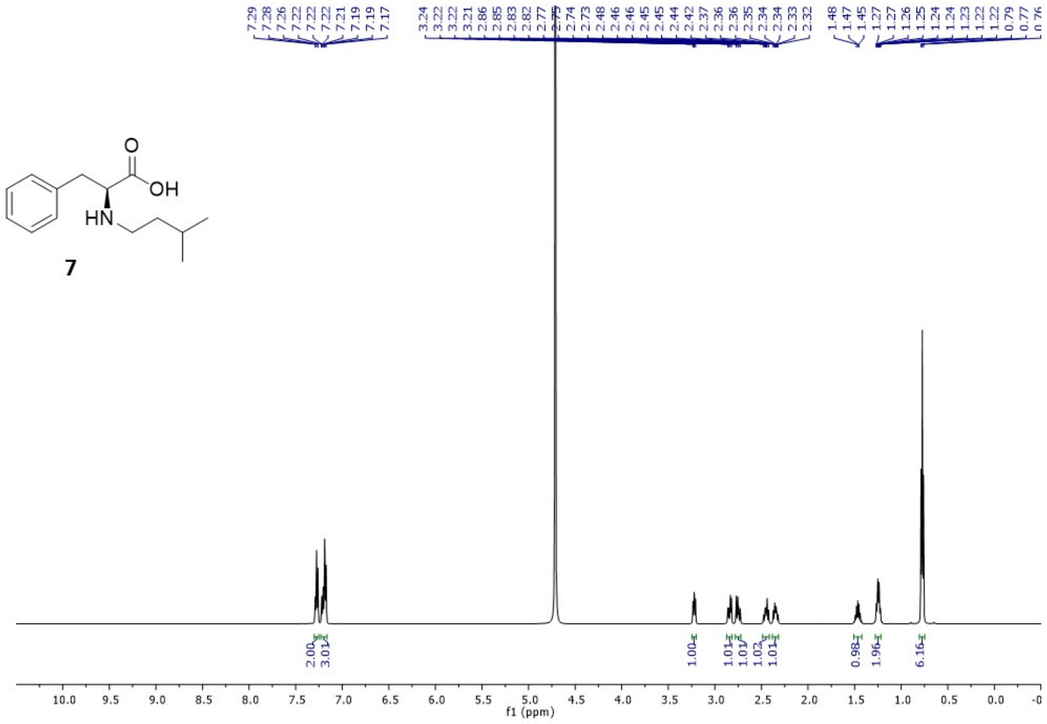

Figure S16. ^{1}H NMR (500 MHz, D_2O-KOH) of isopentyl-L-phenylalanine (**7**).

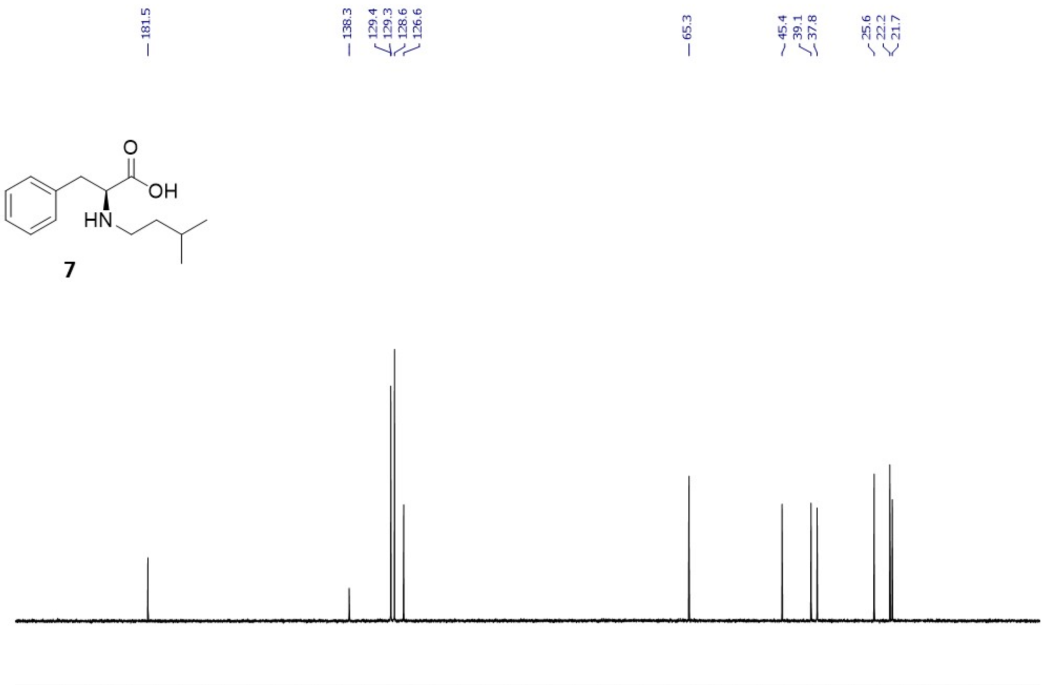

Figure S17. ^{13}C NMR (125 MHz, D_2O-KOH) of isopentyl-L-phenylalanine (**7**).

6. Drug delivery study

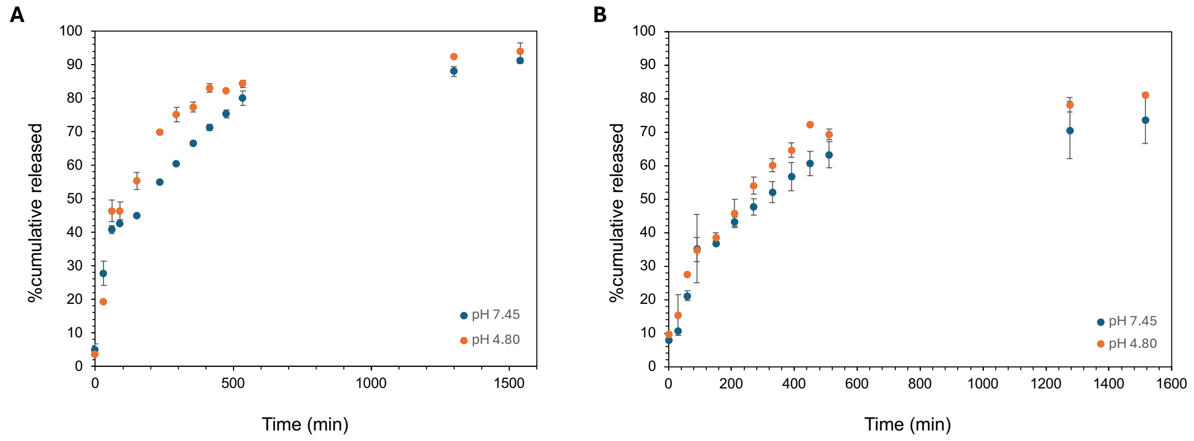

Figure S18. *In vitro* drug release of 5-FU (A) and MTX (B) as a function of pH (4.8) keeping the gelator concentration constant (9.1 g·L^{-1}). Hydrogel was incubated at 37 °C. Aliquots were withdrawn and the same volume was added to the receptor phase. Error bars correspond to the measurement of three replicates (SD = 3).

Table S3. Constant values, correlation coefficient, AIC, and MSC values obtained from equation models studied for the release of 5-FU varying the gelator concentration (8.2 g·L^{-1}) and pH (7.45).

**Molecule**	**Gelator concentration (g/L)**	**pH receptor medium**	**Equation**	**Correlation coefficient (r^2)**	**Parameters and release coefficients**
FU	8.2	7.45	First-order	0.33	AIC: 145<br>MSC: 0.27<br>$K_{F\text{-}O}$: 0.00141
FU	8.2	7.45	Higuchi	-0.016	AIC: 151.6<br>MSC: -0.14<br>K_H: 1.98
FU	8.2	7.45	Korsmeyer-Peppas	0.89	AIC: 117.1<br>MSC: 2.01<br>$K_{K\text{-}P}$: 14.7<br>n: 0.24
**FU**	**8.2**	7.45	**Peppas-Sahlin**	**0.97**	**AIC: 97.8**<br>**MSC: 3.22**<br>**$K1_{P\text{-}S}$: 5.69**<br>**$K2_{P\text{-}S}$: -0.078**<br>**m: 0.45**
**FU**	**8.2**	7.45	**Weibull**	**0.98**	**AIC: 92.6**<br>**MSC: 3.54**<br>**α: 13.9**<br>**β: 0.48**

Table S4. Constant values, correlation coefficient, AIC, and MSC values obtained from equation models studied for the release of 5-FU varying the gelator concentration (9.1 g·L^{-1}) and pH (7.45).

**Molecule**	**Gelator concentration (g/L)**	**pH receptor medium**	**Equation**	**Correlation coefficient (r^2)**	**Parameters and release coefficients**
FU	9.1	7.45	First-order	0.47	AIC: 142.7<br>MSC: 0.52<br>K_{F-O}: 0.00097
FU	9.1	7.45	Higuchi	0.43	AIC: 143.9<br>MSC: 0.44<br>K_H: 1.79
FU	9.1	7.45	Korsmeyer-Peppas	0.82	AIC: 126<br>MSC: 1.57<br>K_{K-P}: 7.45<br>n: 0.32
**FU**	**9.1**	7.45	**Peppas-Sahlin**	**0.97**	**AIC: 100.7**<br>**MSC: 3.14**<br>**$K1_{P-S}$: 4.75**<br>**$K2_{P-S}$: -0.059**<br>**m: 0.45**
**FU**	**9.1**	7.45	**Weibull**	**0.97**	**AIC: 95.5**<br>**MSC: 3.47**<br>**α: 31.5**<br>**β: 0.55**

Table S5. Constant values, correlation coefficient, AIC, and MSC values obtained from equation models studied for the release of 5-FU varying the gelator concentration (9.9 g·L^{-1}) and pH (7.45).

**Molecule**	**Gelator concentration (g/L)**	**pH receptor medium**	**Equation**	**Correlation coefficient (r^2)**	**Parameters and release coefficients**
FU	9.9	7.45	First-order	0.55	AIC: 140.9<br>MSC: 0.67<br>K_{F-O}: 0.00082
FU	9.9	7.45	Higuchi	0.57	AIC: 140.1<br>MSC: 0.72<br>K_H: 1.69
FU	9.9	7.45	Korsmeyer-Peppas	0.71	AIC: 134.6<br>MSC: 1.06<br>K_{K-P}: 3.13<br>n: 0.43
**FU**	**9.9**	7.45	**Peppas-Sahlin**	**0.94**	**AIC: 109**<br>**MSC: 2.66**<br>**$K1_{P-S}$: 4.28**<br>**$K2_{P-S}$: -0.05**<br>**m: 0.45**
**FU**	**9.9**	7.45	**Weibull**	**0.94**	**AIC: 109.7**<br>**MSC: 2.62**<br>**α: 70.1**<br>**β: 0.64**

Table S6. Constant values, correlation coefficient, AIC, and MSC values obtained from equation models studied for the release of MTX varying the gelator concentration (8.2 g·L^{-1}) and pH (7.45).

Molecule	Gelator concentration (g/L)	pH receptor medium	Equation	Correlation coefficient (r^2)	Parameters and release coefficients
MTX	8.2	7.45	First-order	0.39	AIC: 140.8<br>MSC: 0.37<br>$K_{F\text{-}O}$: 0.0007
MTX	8.2	7.45	Higuchi	0.54	AIC: 136.2<br>MSC: 0.66<br>K_H: 1.59
MTX	8.2	7.45	Korsmeyer-Peppas	0.88	AIC: 115.7<br>MSC: 1.94<br>$K_{K\text{-}P}$: 6.50<br>n: 0.32
MTX	**8.2**	7.45	**Peppas-Sahlin**	**0.97**	**AIC: 94.01**<br>**MSC: 3.30**<br>**$K1_{P\text{-}S}$: 3.97**<br>**$K2_{P\text{-}S}$: -0.046**<br>**m: 0.45**
MTX	**8.2**	7.45	**Weibull**	**0.97**	**AIC: 94.9**<br>**MSC: 3.24**<br>**α: 30.2**<br>**β: 0.49**

Table S7. Constant values, correlation coefficient, AIC, and MSC values obtained from equation models studied for the release of MTX varying the gelator concentration (9.1 g·L^{-1}) and pH (7.45).

Molecule	Gelator concentration (g/L)	pH receptor medium	Equation	Correlation coefficient (r^2)	Parameters and release coefficients
MTX	9.1	7.45	First-order	-0.044	AIC: 148.1<br>MSC: -0.16<br>$K_{F\text{-}O}$: 0.0007
MTX	9.1	7.45	Higuchi	0.25	AIC: 142.7<br>MSC: 0.17<br>K_H: 1.62
MTX	9.1	7.45	Korsmeyer-Peppas	0.81	AIC: 121.9<br>MSC: 1.46<br>$K_{K\text{-}P}$: 8.6<br>n: 0.29
MTX	**9.1**	7.45	**Peppas-Sahlin**	**0.96**	**AIC: 99.1**<br>**MSC: 2.89**<br>**$K1_{P\text{-}S}$: 4.45**<br>**$K2_{P\text{-}S}$: -0.057**<br>**m: 0.45**
MTX	**9.1**	7.45	**Weibull**	**0.95**	**AIC: 102.3**<br>**MSC: 2.69**

					**α: 21.4**<br>**β: 0.46**

Table S8. Constant values, correlation coefficient, AIC, and MSC values obtained from equation models studied for the release of MTX varying the gelator concentration (9.9 g·L^{-1}) and pH (7.45).

Molecule	Gelator concentration (g/L)	pH receptor medium	Equation	Correlation coefficient (r^2)	Parameters and release coefficients
MTX	9.9	7.45	First-order	0.49	AIC: 139.9<br>MSC: 0.55<br>K_{F-O}: 0.0007
MTX	9.9	7.45	Higuchi	0.61	AIC: 135.6<br>MSC: 0.83<br>K_H: 1.56
MTX	9.9	7.45	Korsmeyer-Peppas	0.81	AIC: 125.3<br>MSC: 1.47<br>K_{K-P}: 3.75<br>n: 0.39
MTX	**9.9**	7.45	**Peppas-Sahlin**	**0.96**	**AIC: 102.2**<br>**MSC: 2.91**<br>**$K1_{P-S}$: 3.87**<br>**$K2_{P-S}$: -0.043**<br>**m: 0.45**
MTX	**9.9**	7.45	**Weibull**	**0.95**	**AIC: 104.4**<br>**MSC: 2.78**<br>**α: 52.8**<br>**β: 0.57**

Table S9. Constant values, correlation coefficient, AIC, and MSC values obtained from equation models studied for the release of 5-FU keeping the gelator concentration constant (9.1 g·L^{-1}) and varying the pH (4.8).

Molecule	Gelator concentration (g/L)	pH receptor medium	Equation	Correlation coefficient (r^2)	Parameters and release coefficients
FU	8.2	4.8	First-order	0.83	AIC: 124.9<br>MSC: 1.64<br>K_{F-O}: 0.0033
FU	8.2	4.8	Higuchi	-0.28	AIC: 157.2<br>MSC: -0.37<br>K_H: 2.10
FU	8.2	4.8	Korsmeyer-Peppas	0.19	AIC: 150.7<br>MSC: 0.031<br>K_{K-P}: 10.1<br>n: 0.33
**FU**	8.2	4.8	**Peppas-Sahlin**	**0.92**	**AIC: 114.1**<br>**MSC: 2.32**<br>**$K1_{P-S}$: 6.39**

					**$K2_{P-S}$: -0.092**<br>**m: 0.45**
**FU**	8.2	4.8	**Weibull**	**0.98**	**AIC: 86.9**<br>**MSC: 4**<br>**α: 17.7**<br>**β: 0.55**

Table S10. Constant values, correlation coefficient, AIC, and MSC values obtained from equation models studied for the release of MTX keeping the gelator concentration constant (9.1 $g \cdot L^{-1}$) and varying the pH (4.8).

**Molecule**	**Gelator concentration (g/L)**	**pH receptor medium**	**Equation**	**Correlation coefficient (r^2)**	**Parameters and release coefficients**
MTX	9.9	4.8	First-order	0.19	AIC: 146.9<br>MSC: 0.089<br>K_{F-O}: 0.0009
MTX	9.9	4.8	Higuchi	0.22	AIC: 146.4<br>MSC: 0.124<br>K_H: 1.81
MTX	9.9	4.8	Korsmeyer-Peppas	0.82	AIC: 123.9<br>MSC: 1.53<br>K_{K-P}: 11.3<br>n: 0.27
MTX	**9.9**	4.8	**Peppas-Sahlin**	**0.95**	**AIC: 104.0**<br>**MSC: 2.77**<br>**$K1_{P-S}$: 4.97**<br>**$K2_{P-S}$: -0.064**<br>**m: 0.45**
MTX	**9.9**	4.8	**Weibull**	**0.96**	**AIC: 98.9**<br>**MSC: 3.09**<br>**α: 19.7**<br>**β: 0.48**

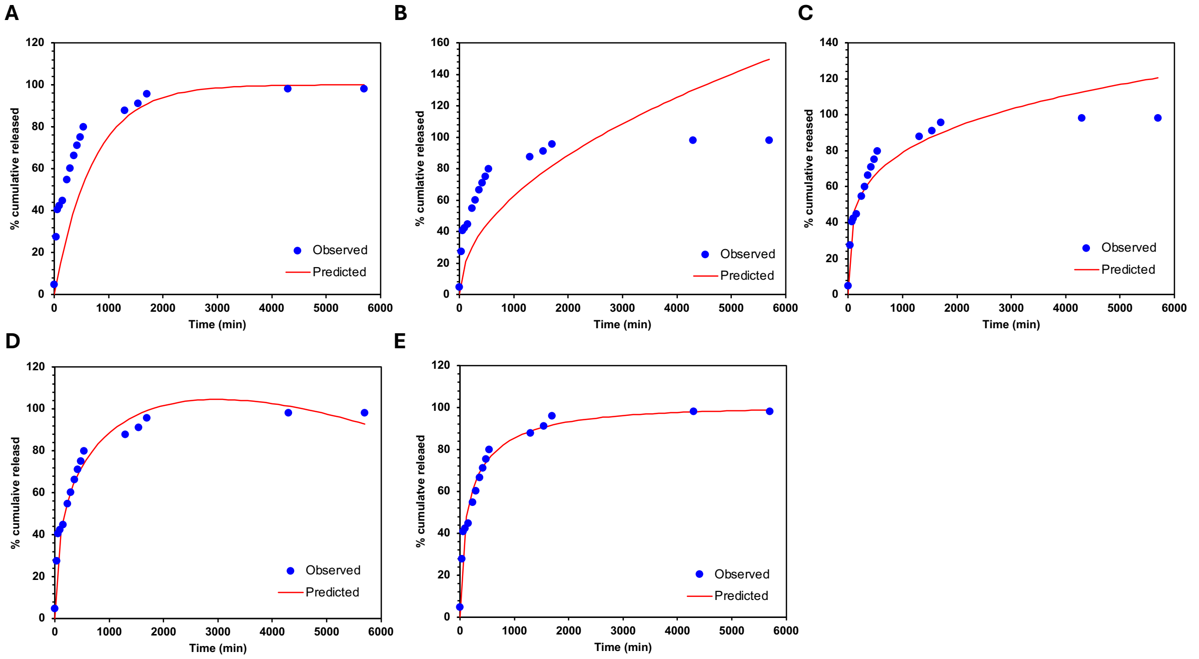

Figure S19. Fitting of 5-FU to First-order (A), Higuchi (B), Korsmeyer-Peppas (C), Peppas-Sahlin (D), and Weibull (E). Blue dots correspond to the experimental data (8.2 g·L^{-1} gelator concentration, PBS as a receptor phase, and pH 7.45). The DDSolver add-in program was used to fit the release data of 5-FU to the models described above. Blue dots correspond to the experimental data and red line corresponds with the predicted correlation to the experimental release.

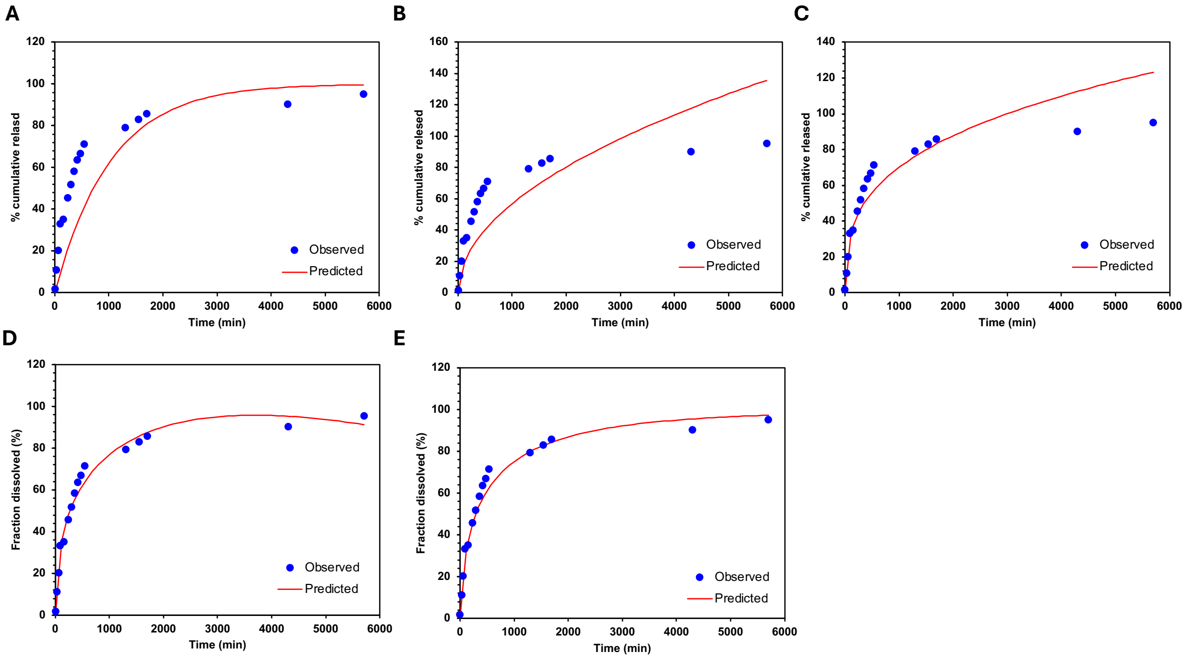

Figure S20. Fitting of 5-FU to First-order (A), Higuchi (B), Korsmeyer-Peppas (C), Peppas-Sahlin (D), and Weibull (E). Blue dots correspond to the experimental data (9.1 g·L^{-1} gelator concentration, PBS as a receptor phase, and pH 7.45). The DDSolver add-in program was used to fit the release data of 5-FU to the models described above. Blue dots correspond to the experimental data and red line corresponds with the predicted correlation to the experimental release.

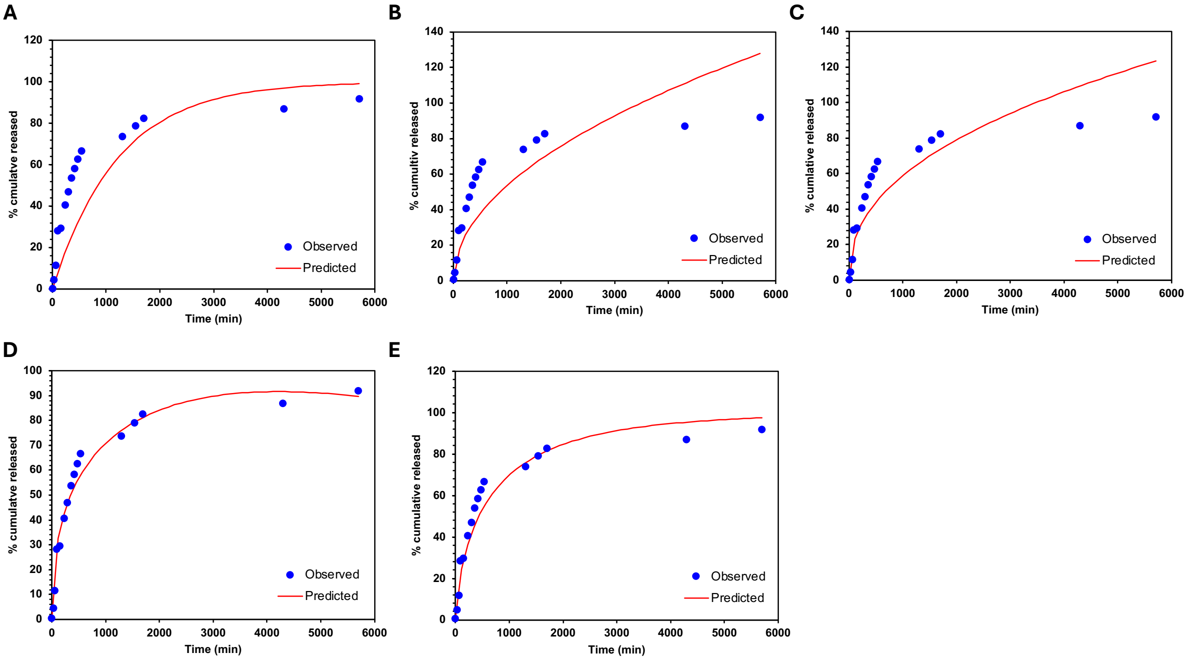

Figure S21. Fitting of 5-FU to First-order (A), Higuchi (B), Korsmeyer-Peppas (C), Peppas-Sahlin (D), and Weibull (E). Blue dots correspond to the experimental data (9.9 g·L^{-1} gelator concentration, PBS as a receptor phase, and pH 7.45). The DDSolver add-in program was used to fit the release data of 5-FU to the models described above. Blue dots correspond to the experimental data and red line corresponds with the predicted correlation to the experimental release.

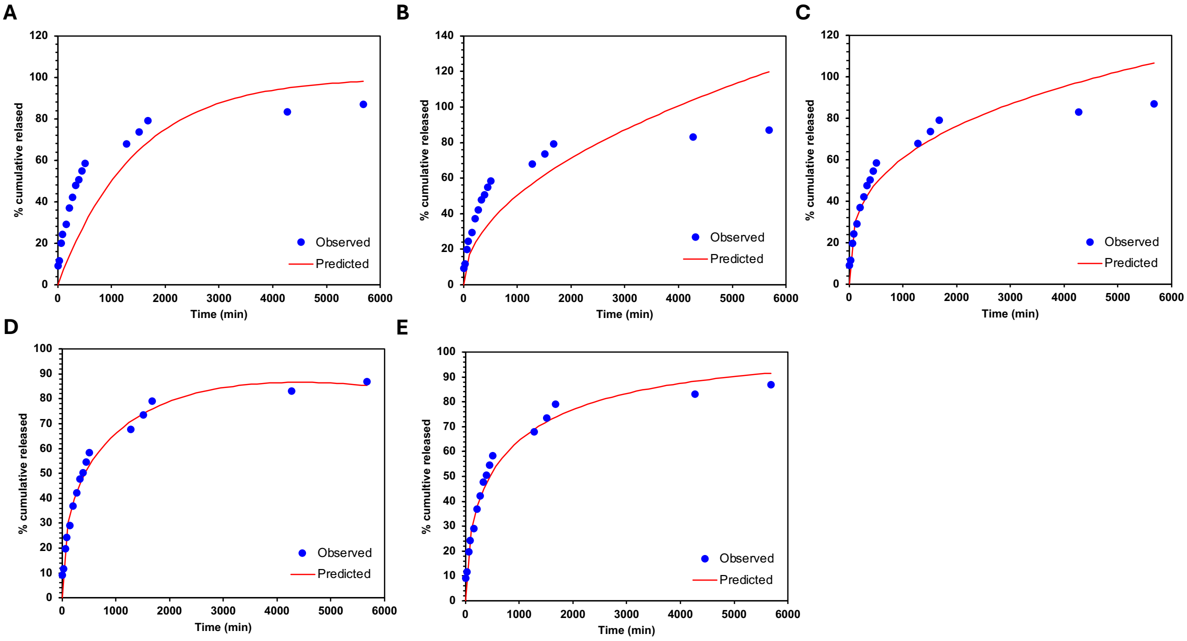

Figure S22. Fitting of MTX to First-order (A), Higuchi (B), Korsmeyer-Peppas (C), Peppas-Sahlin (D), and Weibull (E). Blue dots correspond to the experimental data (8.2 g·L-1 gelator concentration, PBS as a receptor phase, and pH 7.45). The DDSolver add-in program was used to fit the release data of MTX to the models described above. Blue dots correspond to the experimental data and red line corresponds with the predicted correlation to the experimental release.

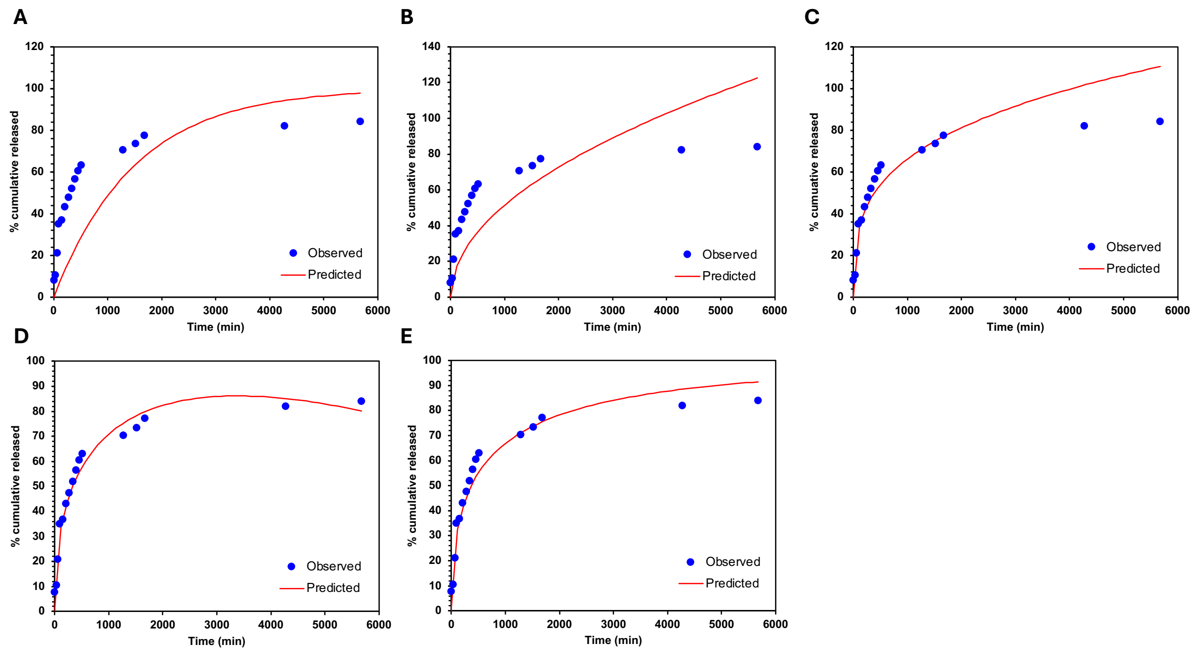

Figure S23. Fitting of MTX to First-order (A), Higuchi (B), Korsmeyer-Peppas (C), Peppas-Sahlin (D), and Weibull (E). Blue dots correspond to the experimental data (9.1 g·L^{-1} gelator concentration, PBS as a receptor phase, and pH 7.45). The DDSolver add-in program was used to fit the release data of MTX to the models described above. Blue dots correspond to the experimental data and red line corresponds with the predicted correlation to the experimental release.

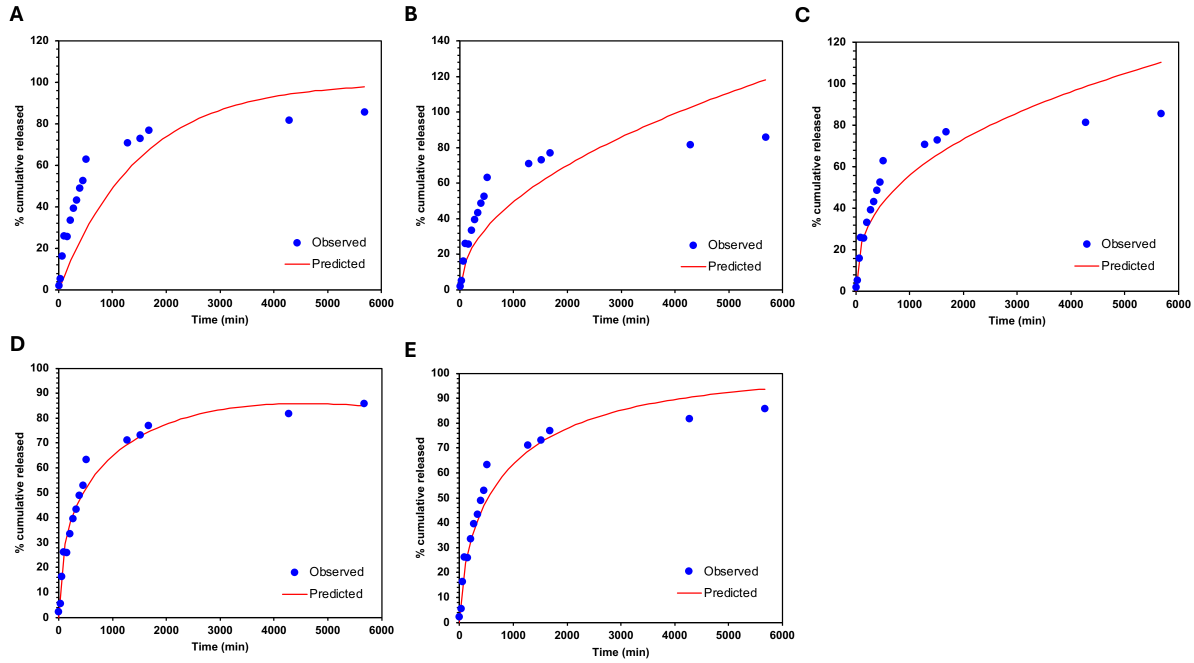

Figure S24. Fitting of MTX to First-order (A), Higuchi (B), Korsmeyer-Peppas (C), Peppas-Sahlin (D), and Weibull (E). Blue dots correspond to the experimental data (9.9 g·L^{-1} gelator concentration, PBS as a receptor phase, and pH 7.45). The DDSolver add-in program was used to fit the release data of MTX to the models described above. Blue dots correspond to the experimental data and red line corresponds with the predicted correlation to the experimental release.

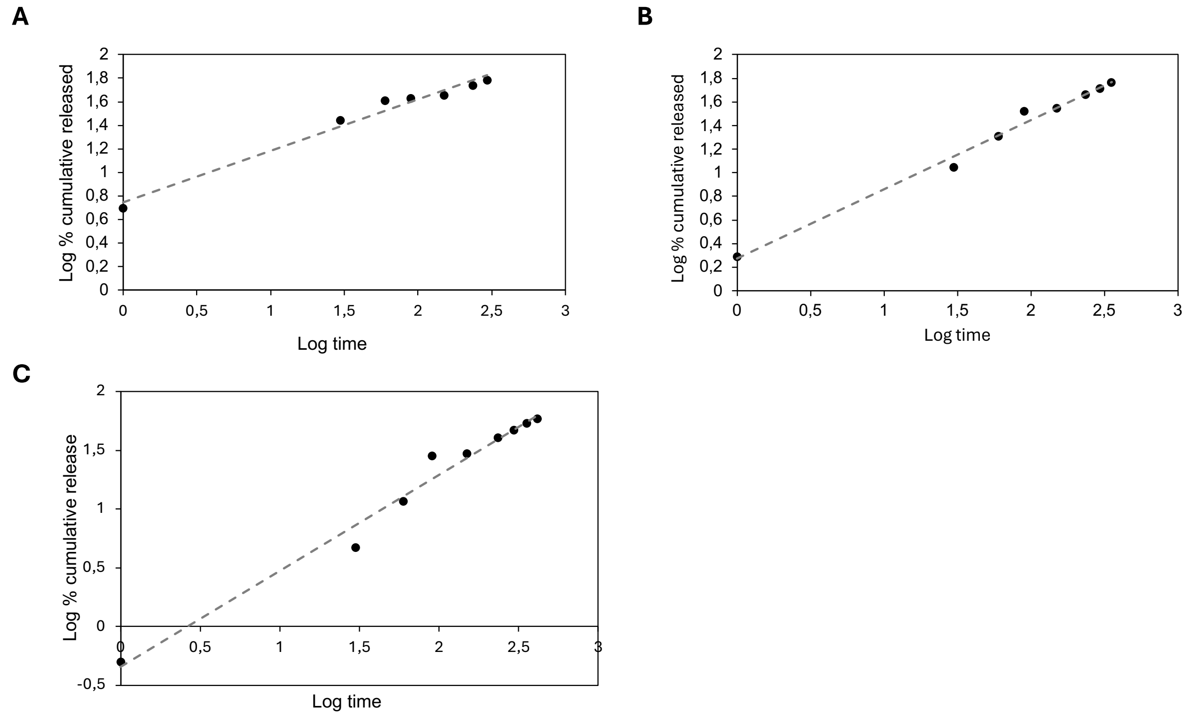

Figure S25. Log-log plot of 5-FU released versus time varying the gelator concentration (A) 8.2 g·L^{-1}; (B) 9.1 g·L^{-1}; and (C) 9.9 g·L^{-1}. The first 60% of drug released was considered in this study.

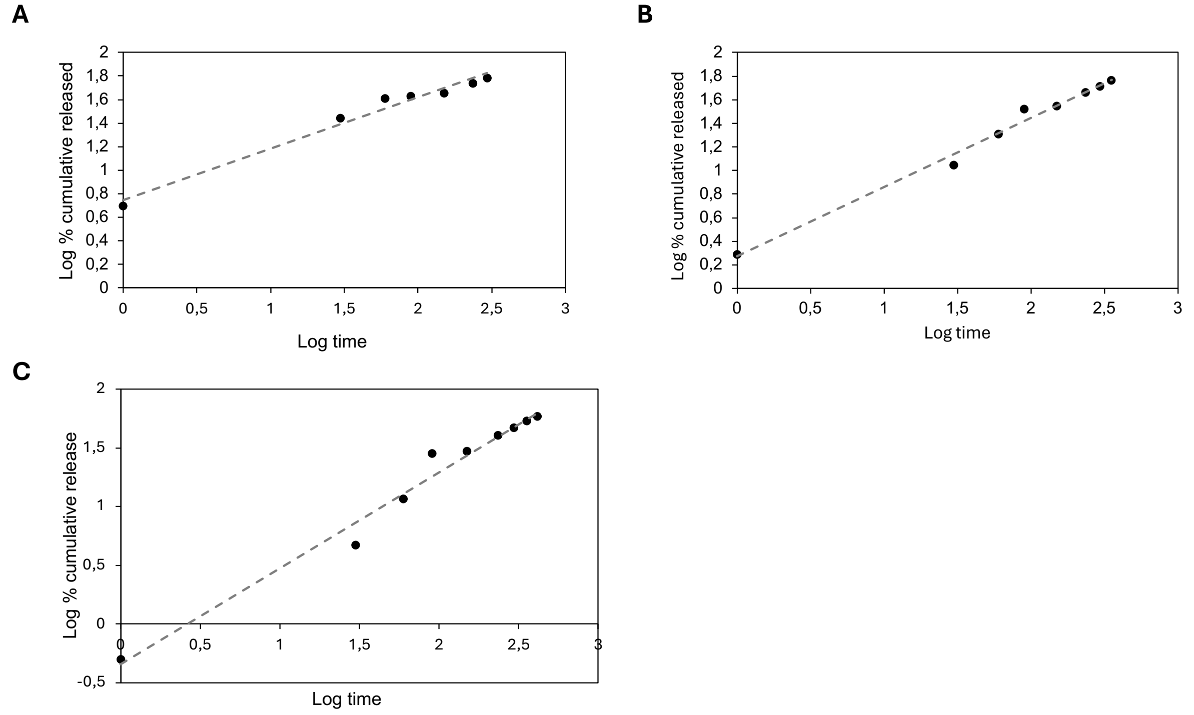

Figure S26. Log-log plot of MTX released versus time varying the gelator concentration (A) 8.2 g·L^{-1}; (B) 9.1 g·L^{-1}; and (C) 9.9 g·L^{-1}. The first 60% of drug released was considered in this study.

1. Kumar, N.; Khullara, S.; Mandal, S. K. Controlling the self-assembly of homochiral coordination architectures of CuII by the substitution in amino acid based ligands: synthesis, crystal structures and physicochemical properties. *Dalton Trans*. **2015**, *44*, 5672–5687.
2. Bermejo-López, A.; Raeder, M.; Martínez-Castro, E.; Martín-Matute, B. Selective and quantitative functionalization of unprotected α-amino acids using a recyclable homogeneous catalyst. *Chem* **2022**, *8*, 3302–3323.
3. Wei, C.-W.; Wang, X.-J.; Gao, S.-Q.; Wen, G.-B.; Lin, Y.-W. A Phenylalanine derivative containing a 4-pyridine group can construct both single crystals and a selective Cu-Ag bimetallohydrogel. *Eur. J. Inorg. Chem.* **2019**, 1349–1353.
